# Supplementary material for: Geochemical studies on rock varnish and petroglyphs in the Owens and Rose Valleys, California
Source: PLoS One. 2020 Aug 5;15(8):e0235421. doi: 10.1371/journal.pone.0235421 (PMC7405993; doi:10.1371/journal.pone.0235421)
Supplement: S1 Table — (PDF) [file pone.0235421.s001.pdf]

S1 Table. Factors other than time known to influence varnish growth on petroglyphs.

Climate and microclimate	Moisture: There is a maximum growth rate at intermediate aridity. Both, hyperarid and humid conditions prevent and/or reverse varnish growth. Repeated moistening by dew, fog, drizzle, etc. enhances growth. Enhanced moisture in canyons or dry wash beds can locally enhance varnish growth.
	Temperature: Can affect weathering and oxidation rates, as well as microbial activity.
	Wind: High winds can lead to aeolian erosion of varnish. Cold-air drainage is important in steep terrain.
	Exposure: Erosion, dust deposition, and surface moisture are influenced by cardinal orientation and inclination of the rock surface, as well as by small-scale topography.
	Surface runoff: Water running off over cliff edges can strongly enhance varnish growth, often resulting in obvious streaking. Shielding by overhanging cliffs or other factors producing rain shadow can reduce varnish growth.
Substrate characteristics	Stability: Resistance of rock substrate against weathering and erosion facilitates formation of a thick and long-lived varnish.
	Roughness and porosity: A rough and porous surface can retain dust and moisture better than a smooth one, facilitating varnish development. An effect of thermal conductivity of the host rock is also possible.
	Microtopography: Small depressions on upper surfaces of boulders (microbasins) retain dust and moisture and promote thick and layered varnish.
	Iron mineral content of the substrate rock may affect initial varnish growth by catalysis.
Geochemical variables	Physicochemical conditions: Varnish formation only occurs in a limited range of pH and Eh. Biogenic organic acids can dissolve varnish.
	Dust flux: Amount and composition of deposited dust regulate the supply of Mn and other trace elements.
	Air pollution: Especially in urban and near-urban conditions, trace metal supply by air pollution can strongly enhance varnish growth.
Microbiology	The amount and species composition of the microbial community on the rock surface can affect both varnish formation and weathering. Microcolonial fungi can dissolve varnish.
Petroglyph-specific issues	Residual varnish: If the original varnish was not completely removed during the creation of the petroglyph, residual varnish can bias the measurements causing spurious regrowth estimates.
	Reworking: Petroglyphs are sometimes reworked at later times, resulting in less apparent regrowth.
	Change in growth rate with time: Evidence suggests that varnish grows more rapidly right after petroglyph creation and eventually comes to a slowdown and halt.
	Change in surface characteristics: Petroglyph creation by abrasion or pecking changes the surface roughness and texture, influencing varnish growth rate
