# Supplementary material for: Geochemical studies on rock varnish and petroglyphs in the Owens and Rose Valleys, California
Source: PLoS One. 2020 Aug 5;15(8):e0235421. doi: 10.1371/journal.pone.0235421 (PMC7405993; doi:10.1371/journal.pone.0235421)
Supplement: S1 Fig — (Map services and data available from U.S. Geological Survey, National Geospatial Program.) (PDF) [file pone.0235421.s002.pdf]

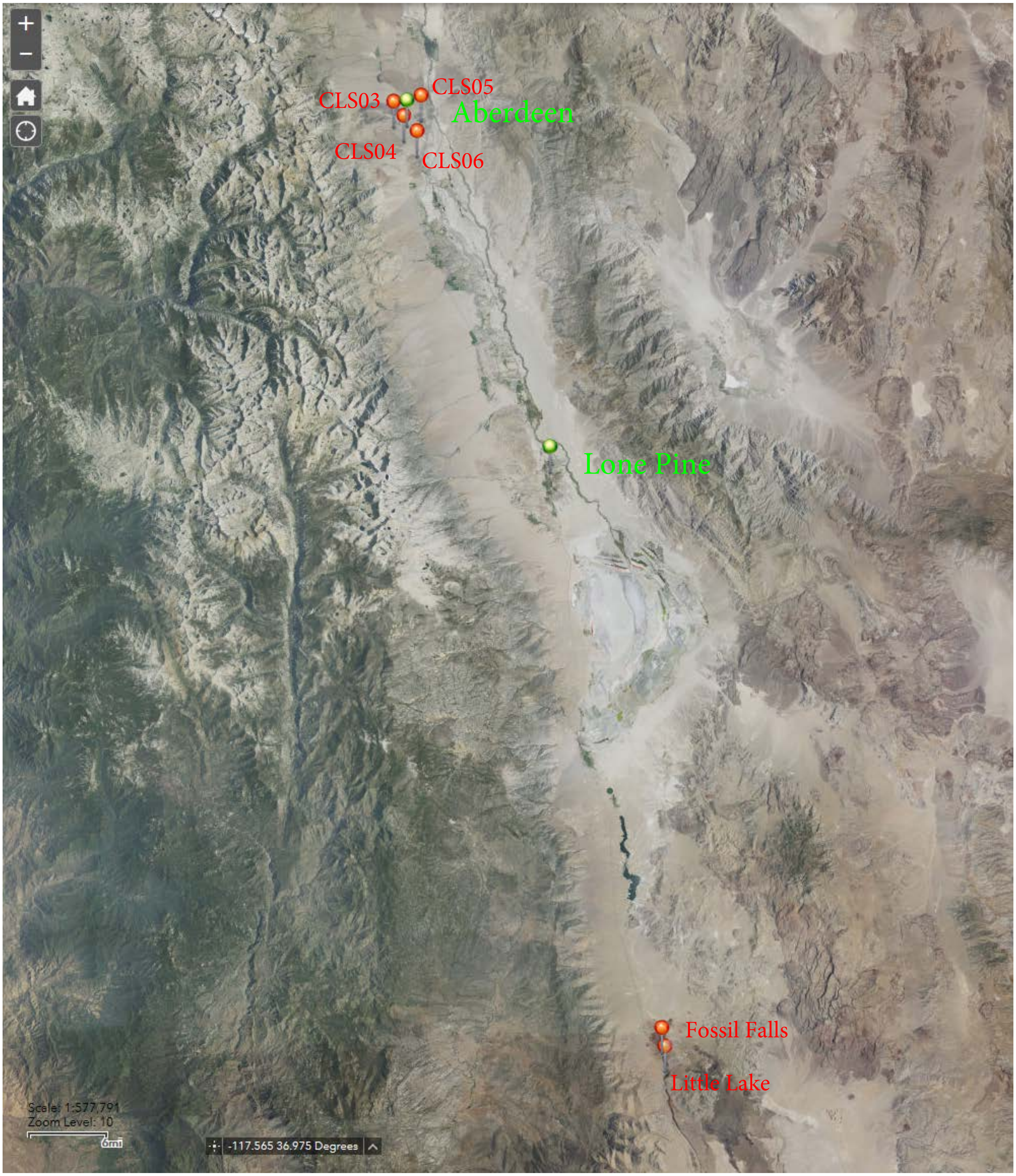

S2 Figure. Overview map of the study area in Owens and Rose Valleys. (Map services and data available from U.S. Geological Survey, National Geospatial Program.)
