# Supplementary material for: Geochemical studies on rock varnish and petroglyphs in the Owens and Rose Valleys, California
Source: PLoS One. 2020 Aug 5;15(8):e0235421. doi: 10.1371/journal.pone.0235421 (PMC7405993; doi:10.1371/journal.pone.0235421)

S3 Figure

Images of the petroglyphs measured in Rose valley

LLH-1

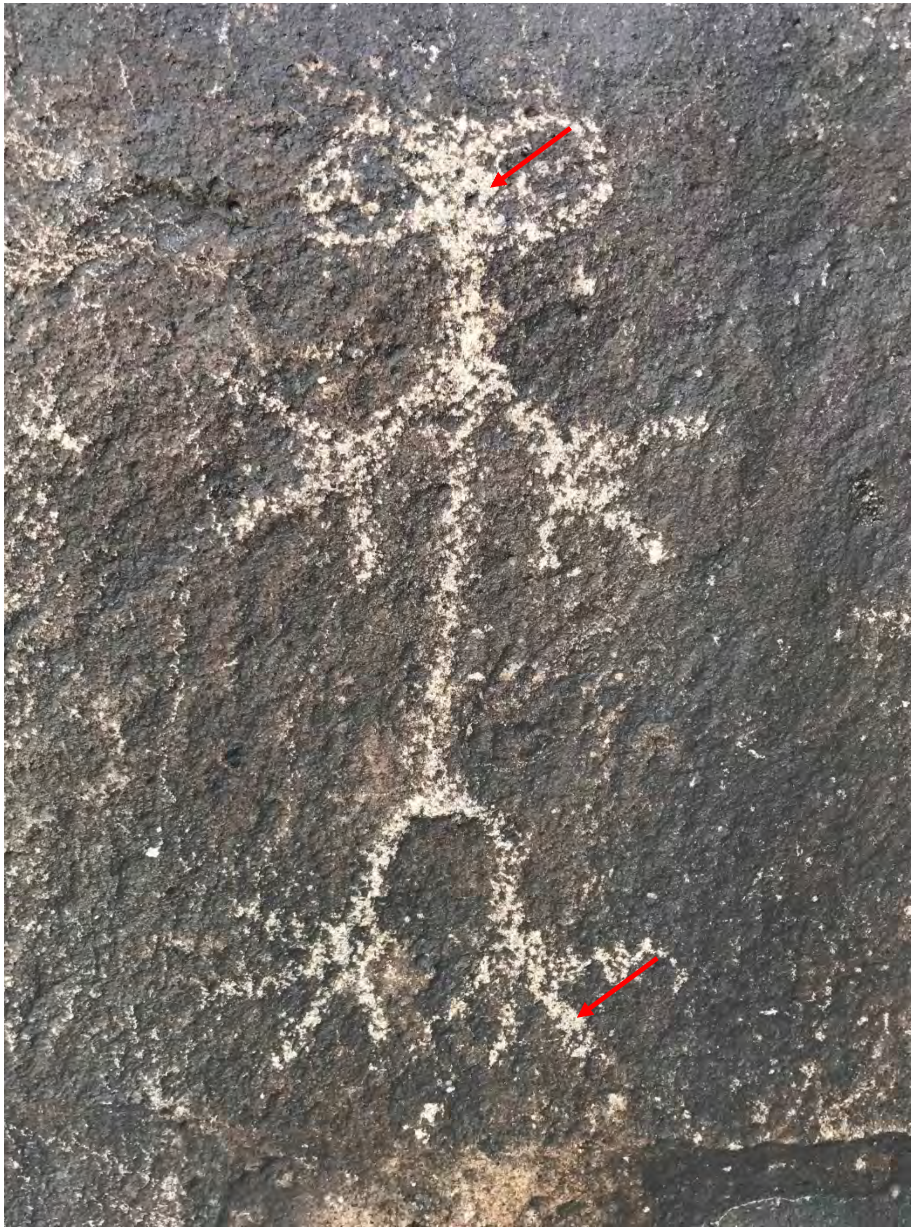

LLH-2

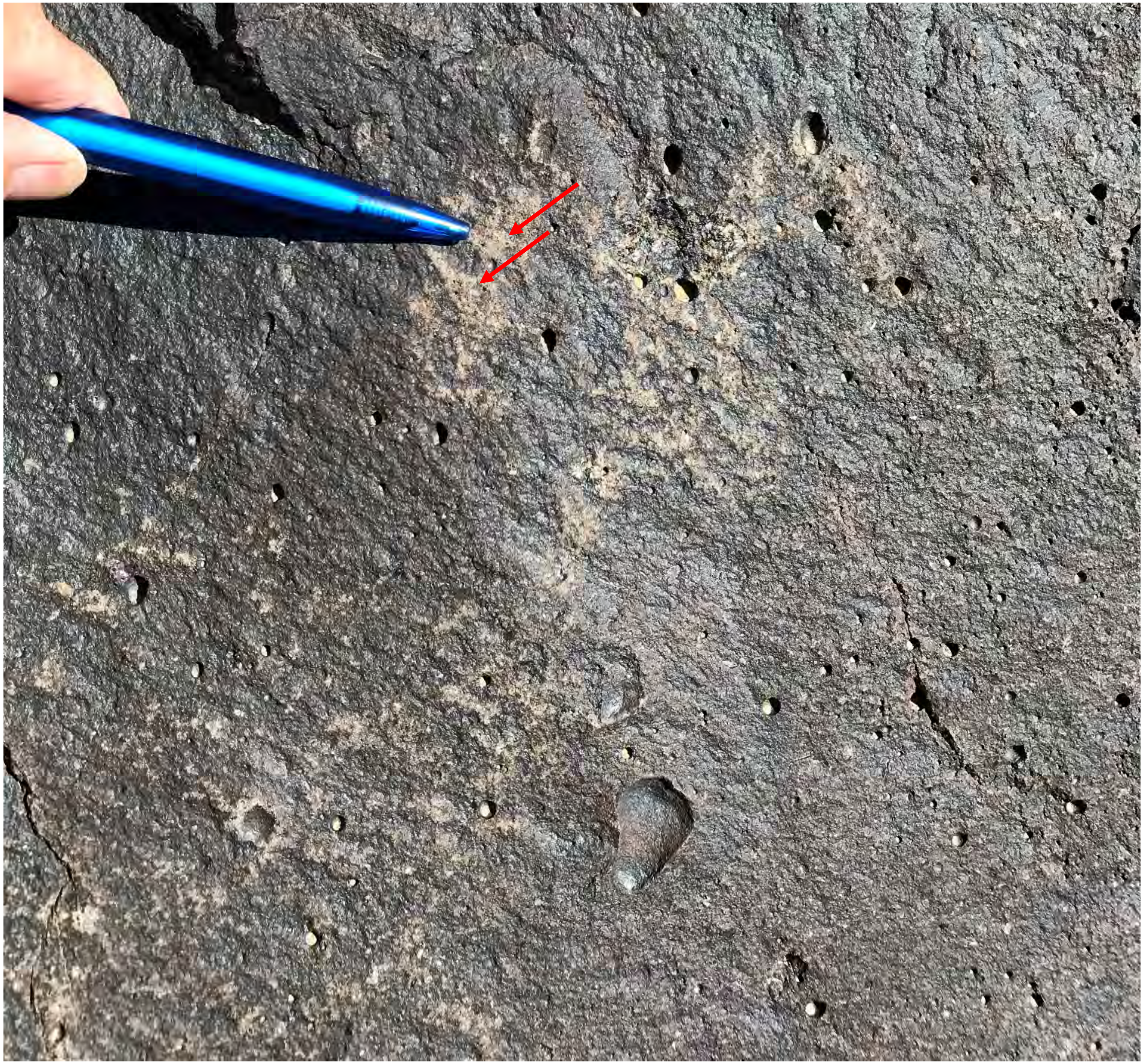

LLH-3

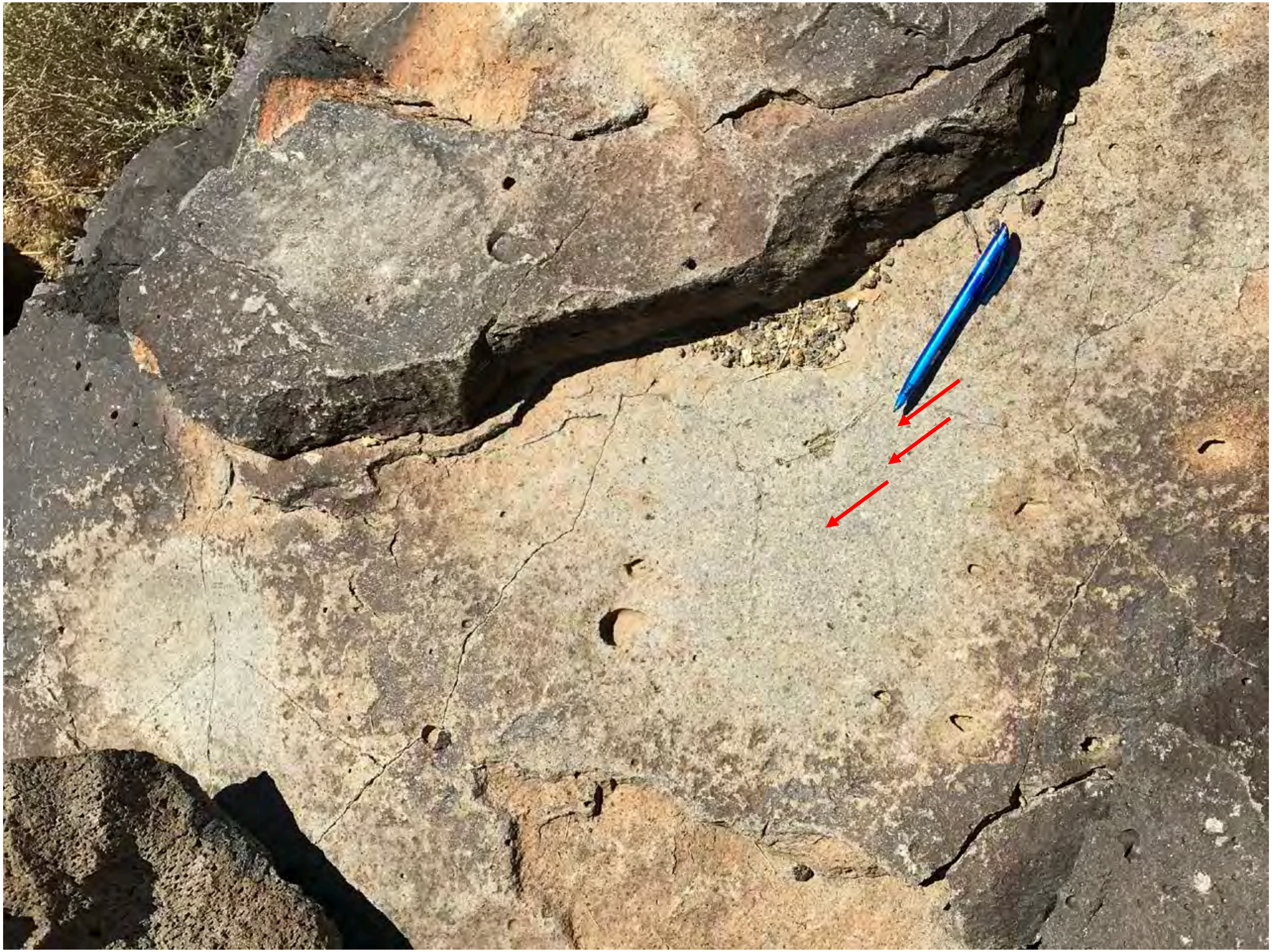

FFS-1

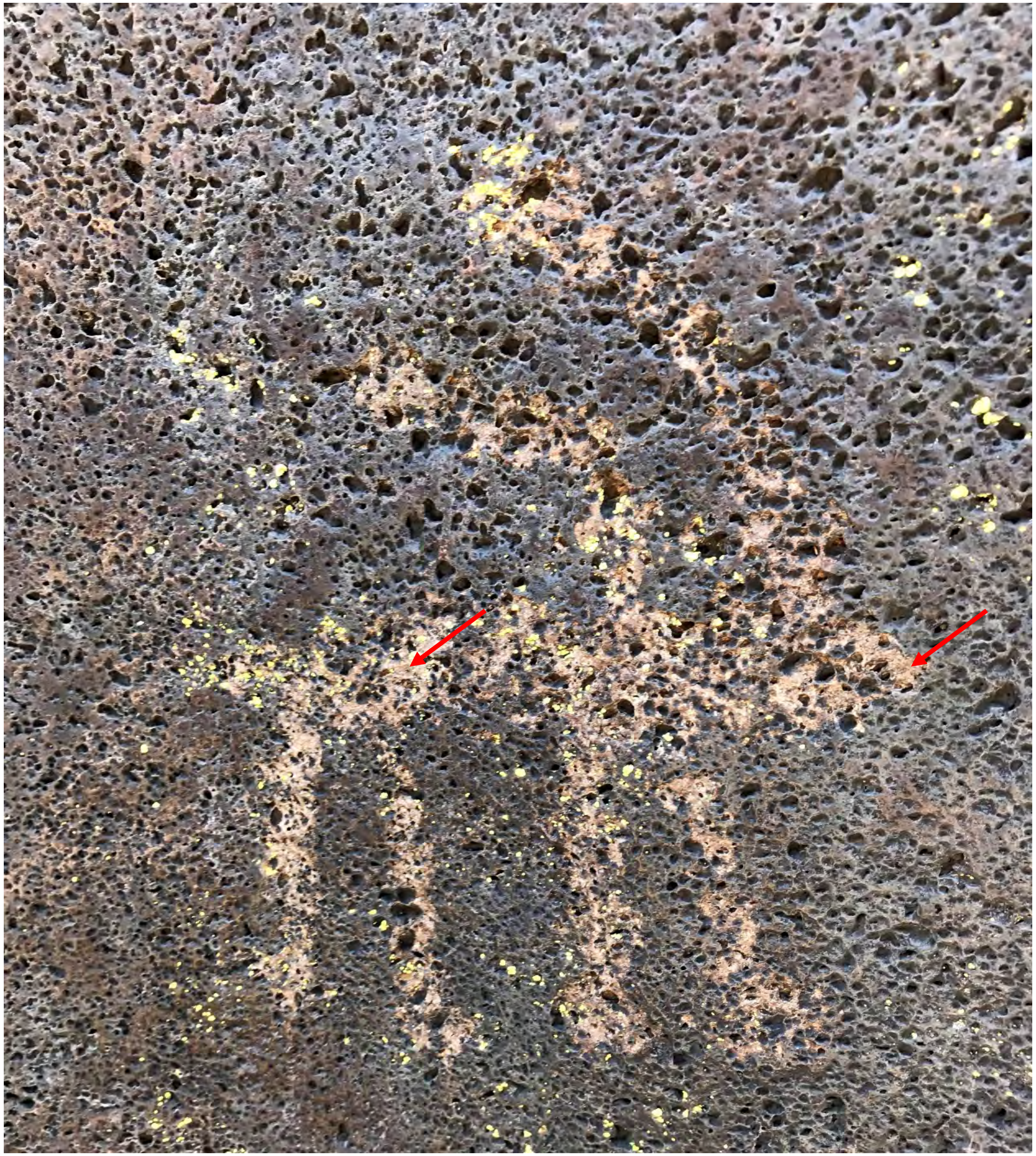

FFS-2

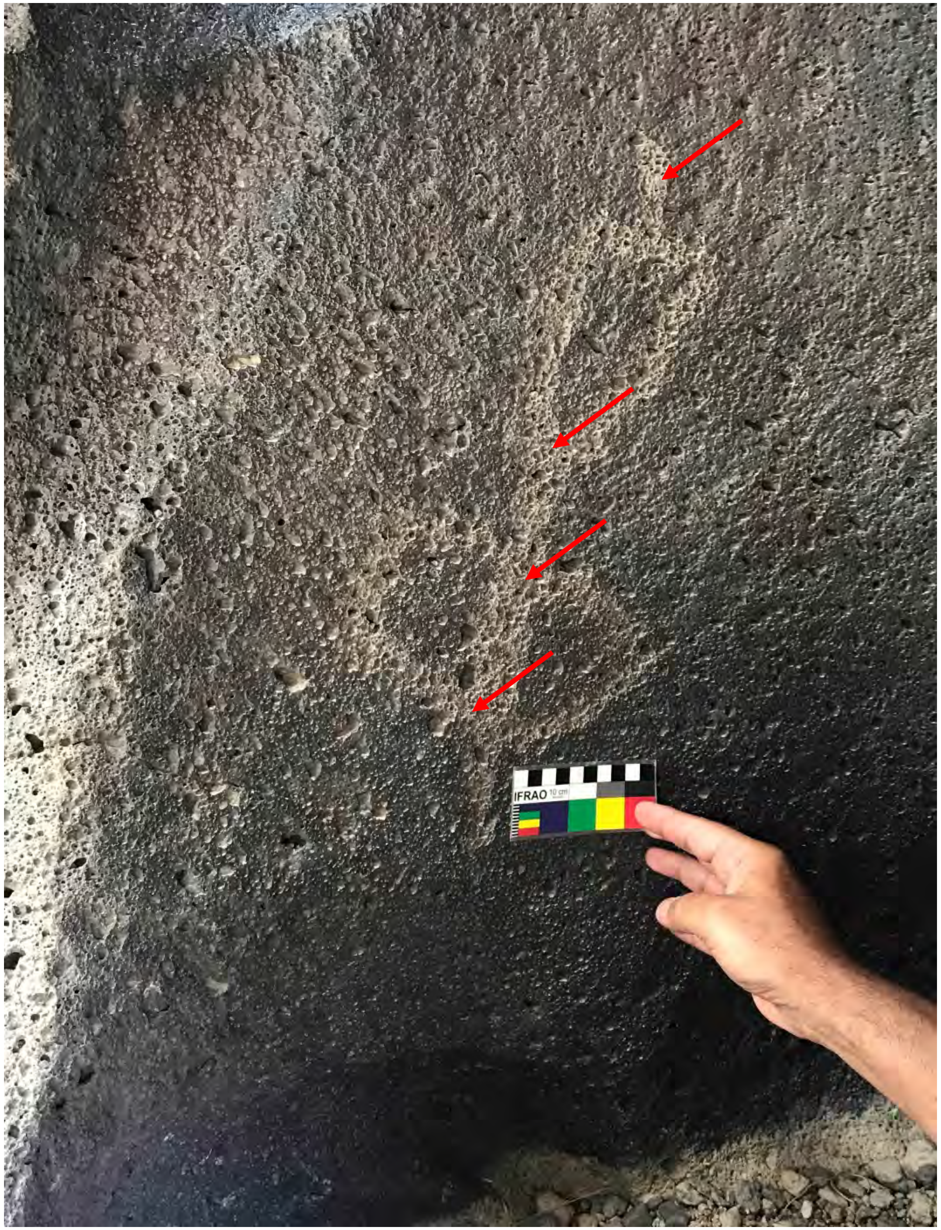

FF-1, FF-2, and FF-3

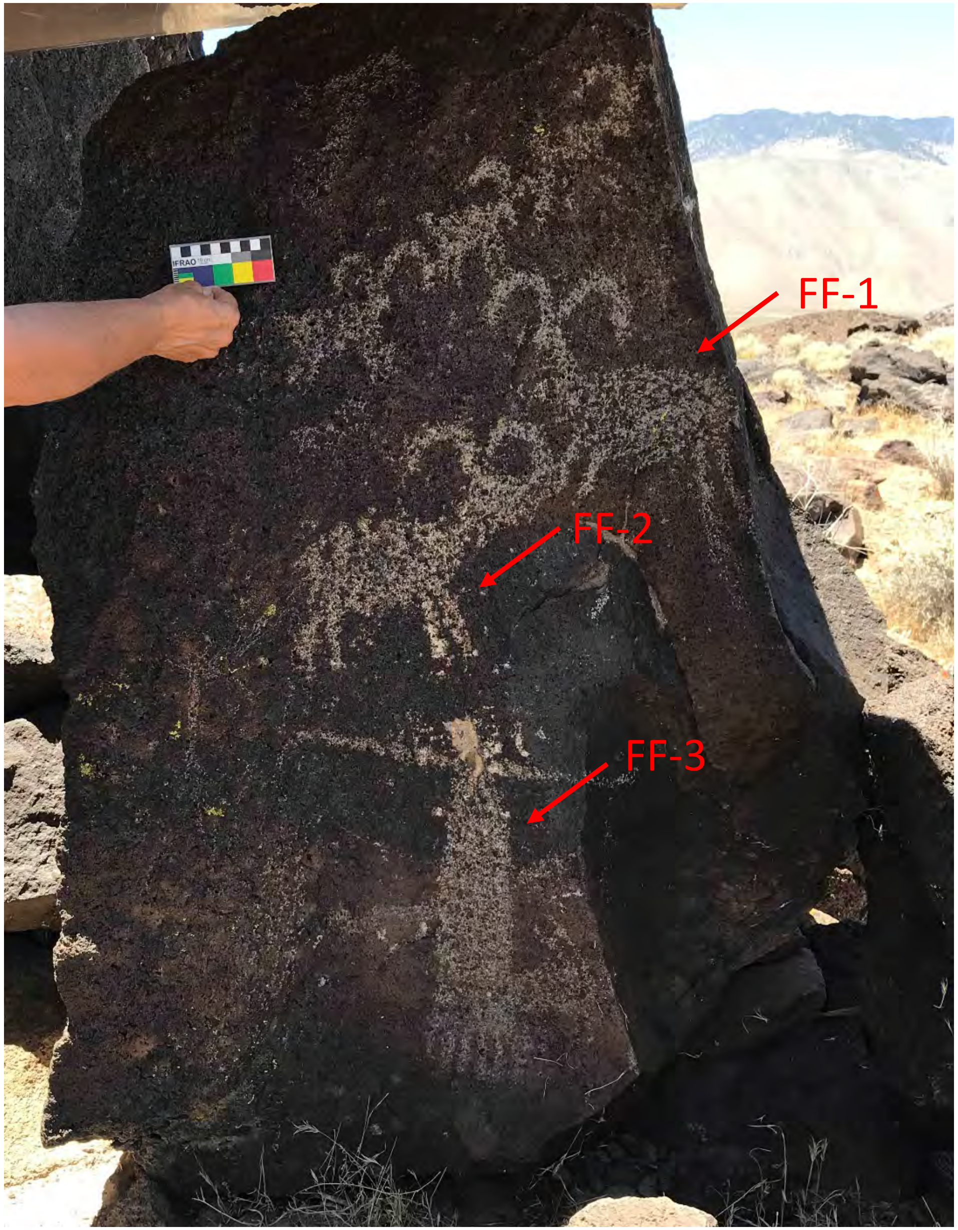

FF-1 and FF-2

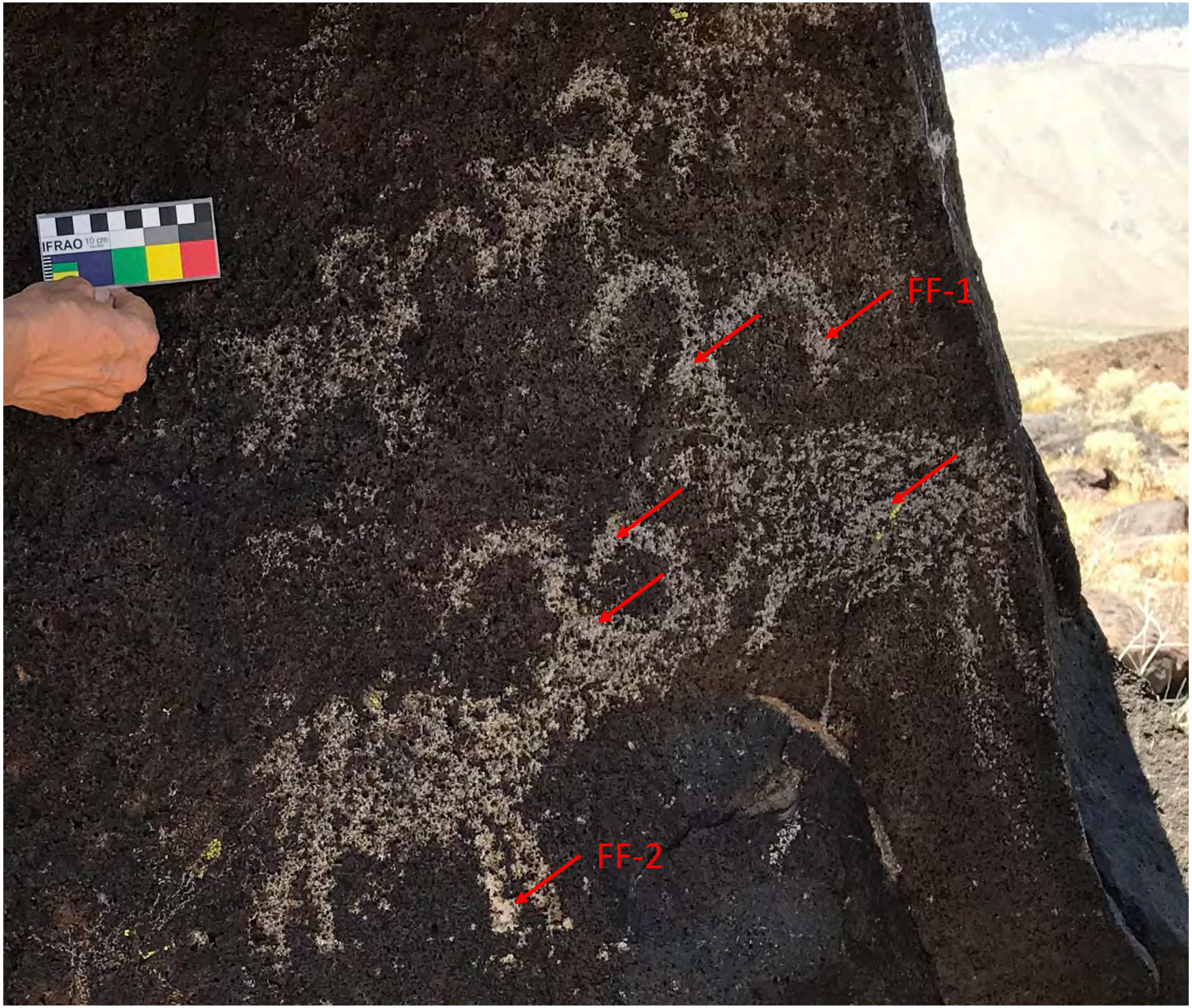

FF-3

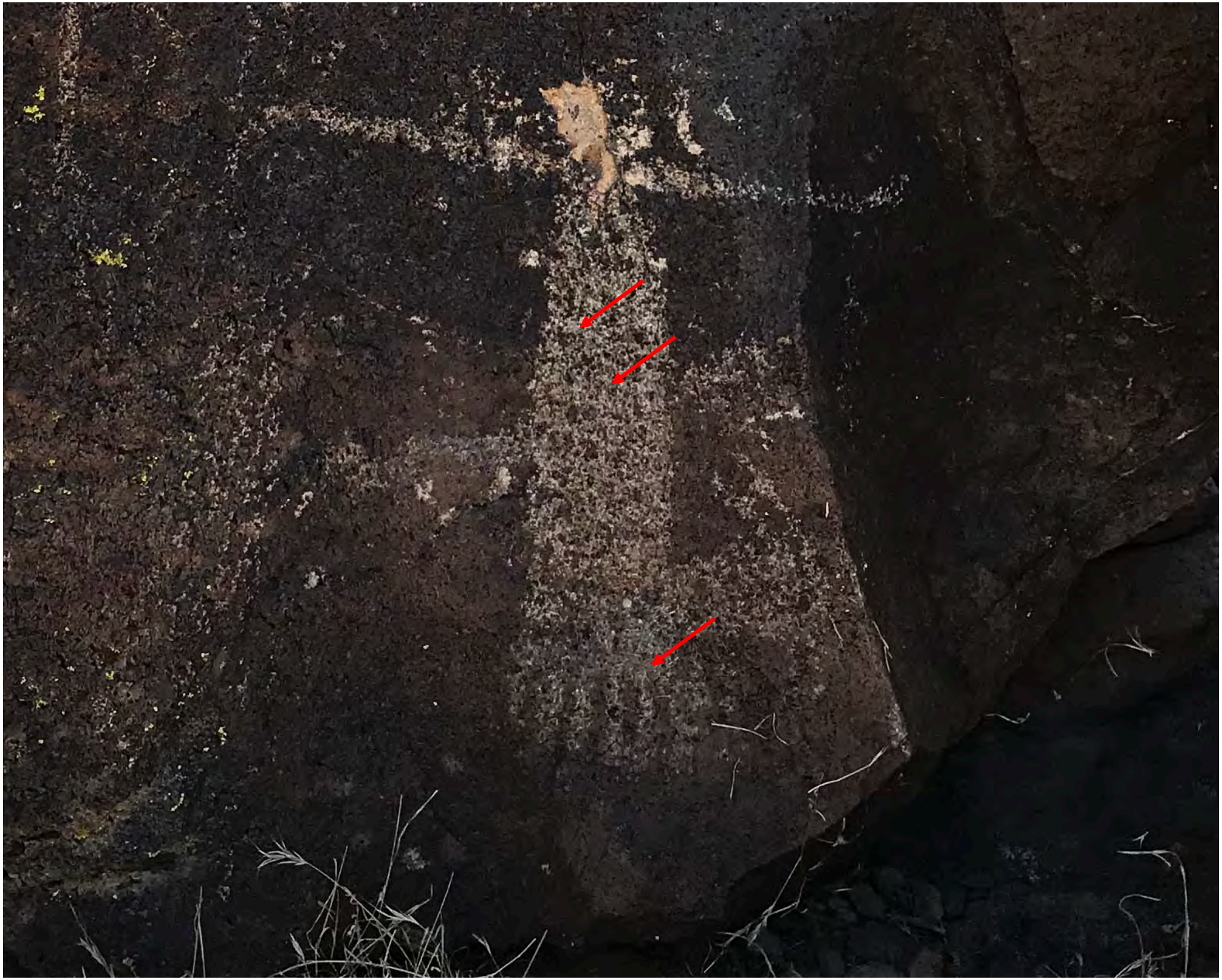

LLA-1

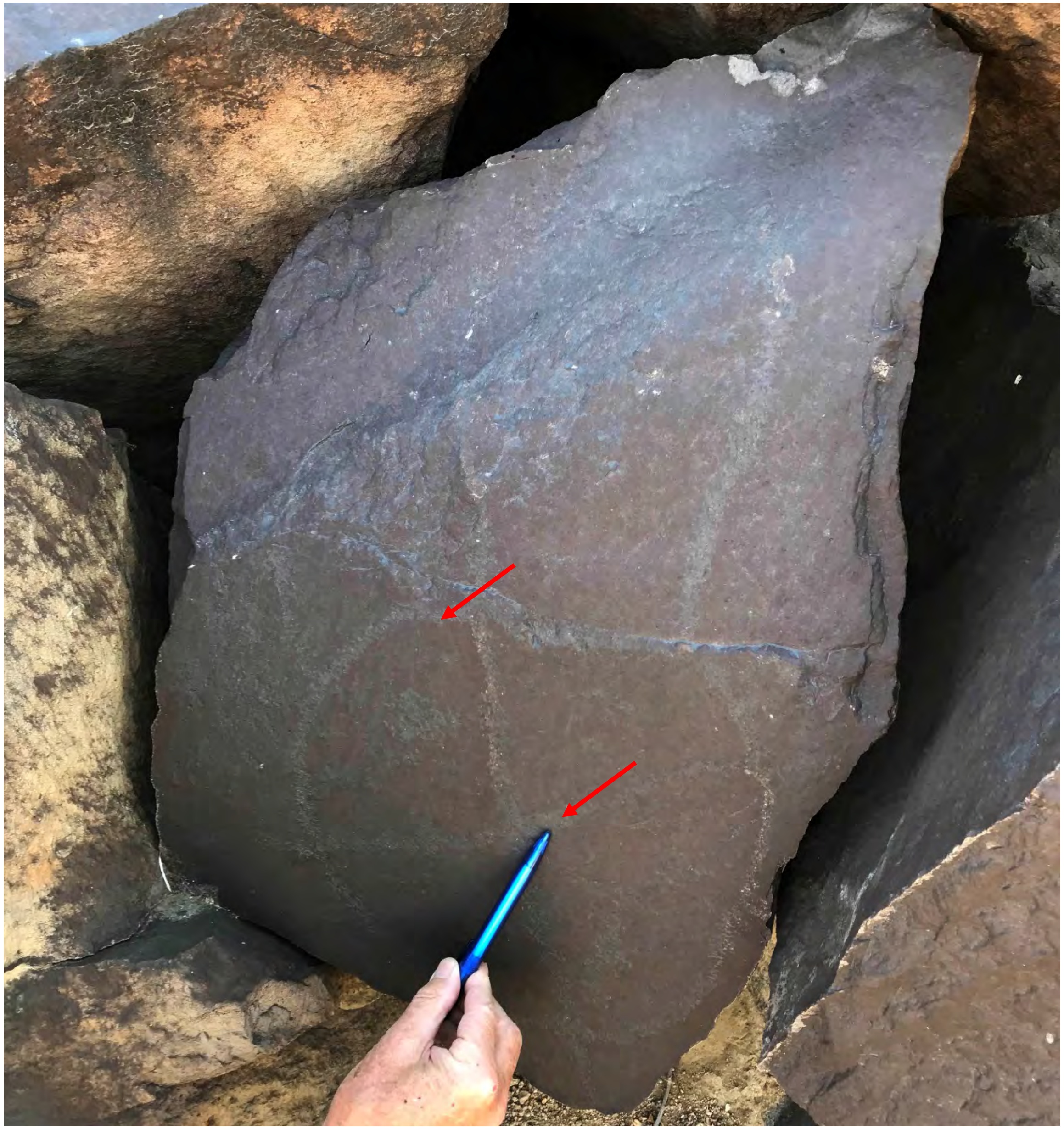

LLA-2 and LLA-3

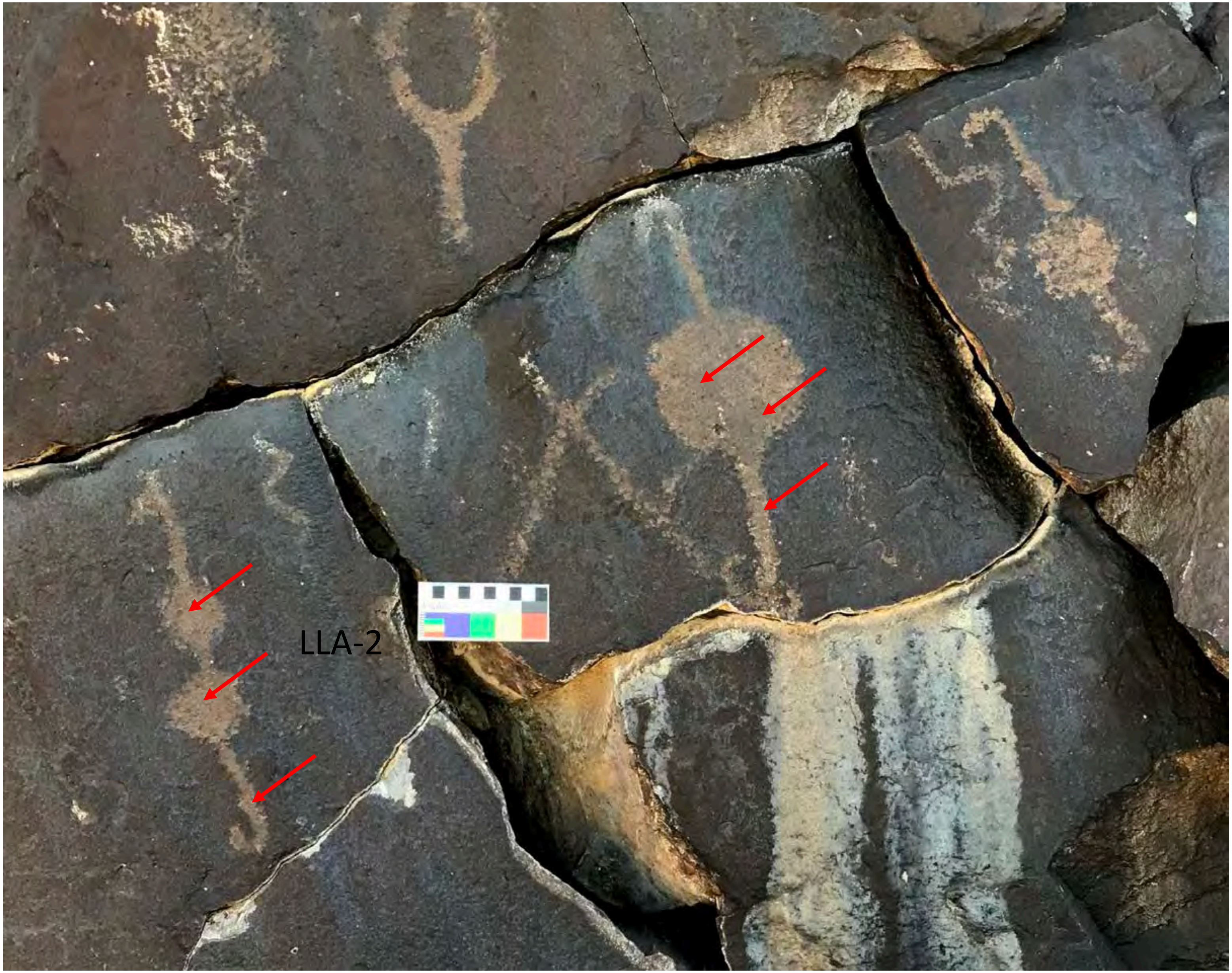

LLA-4

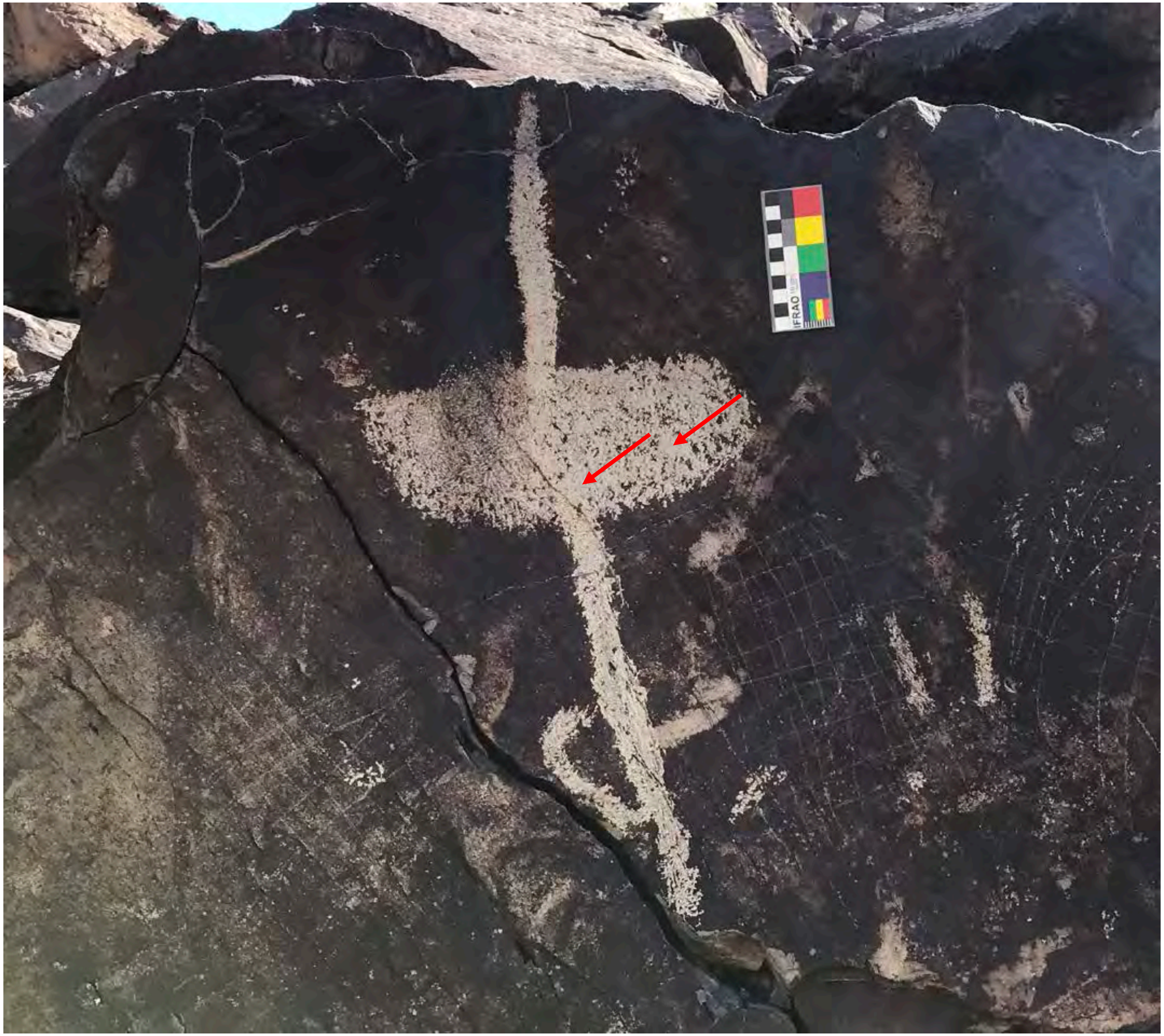

LL8-1

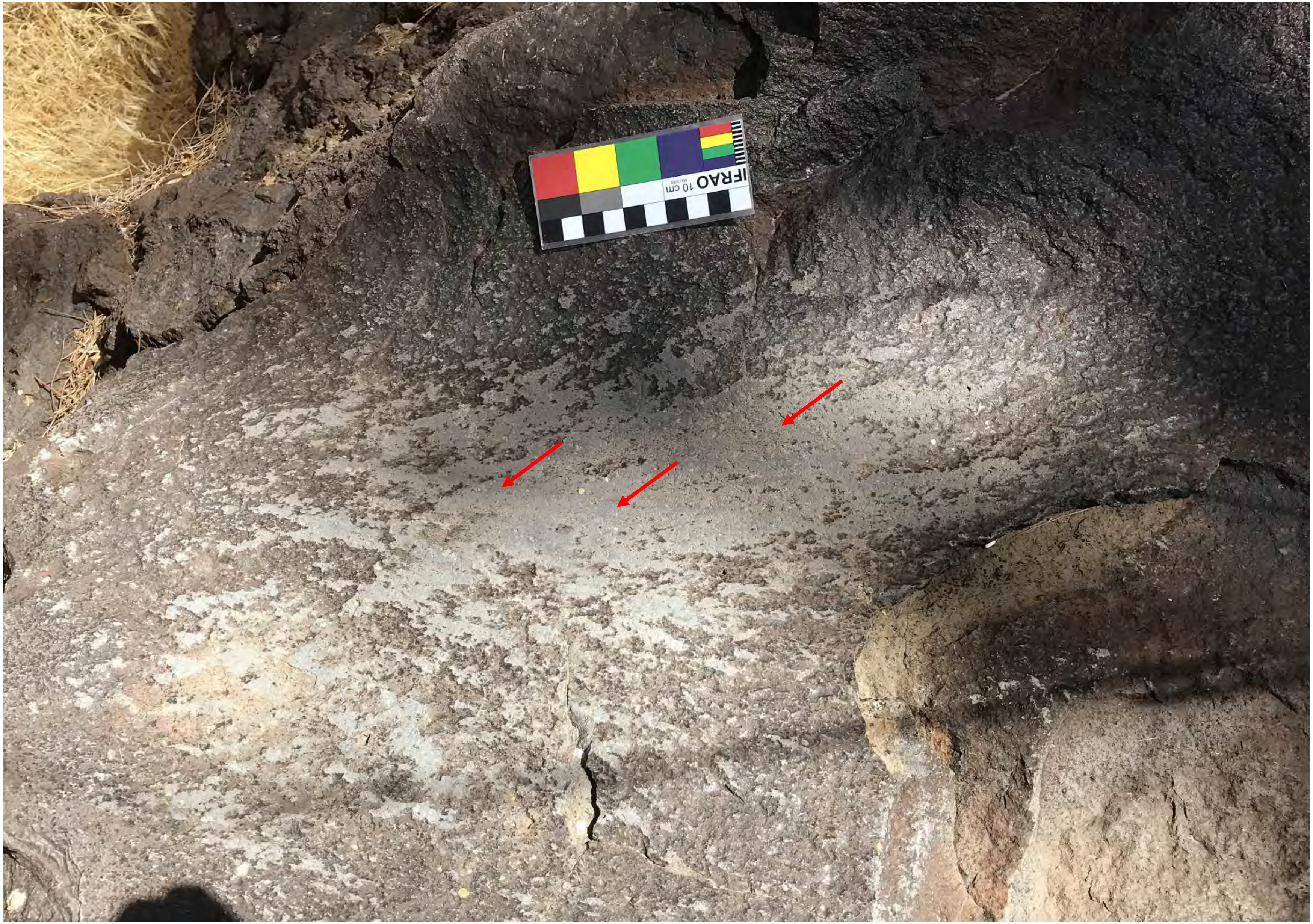

LL8-2

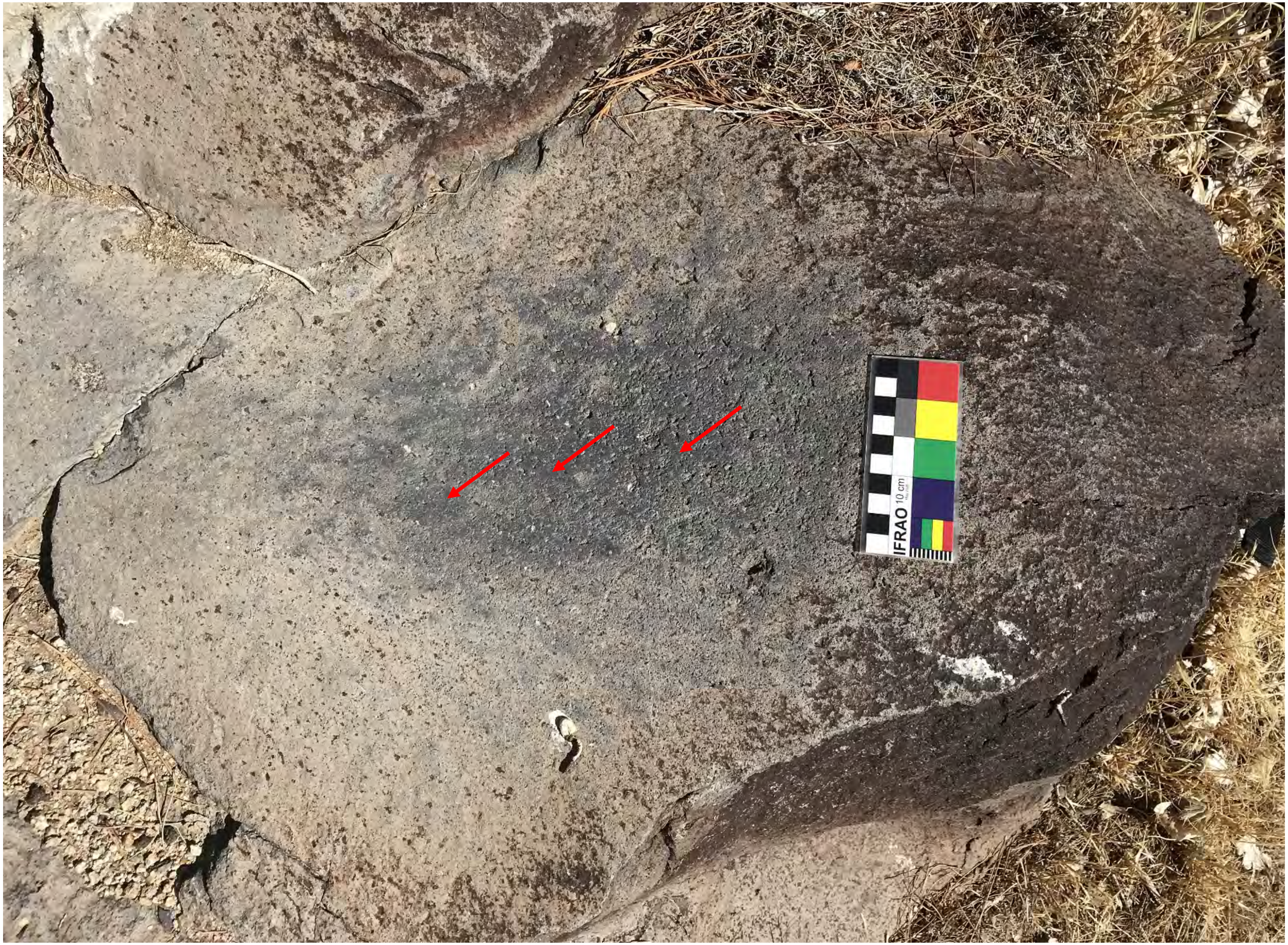

LL8-3

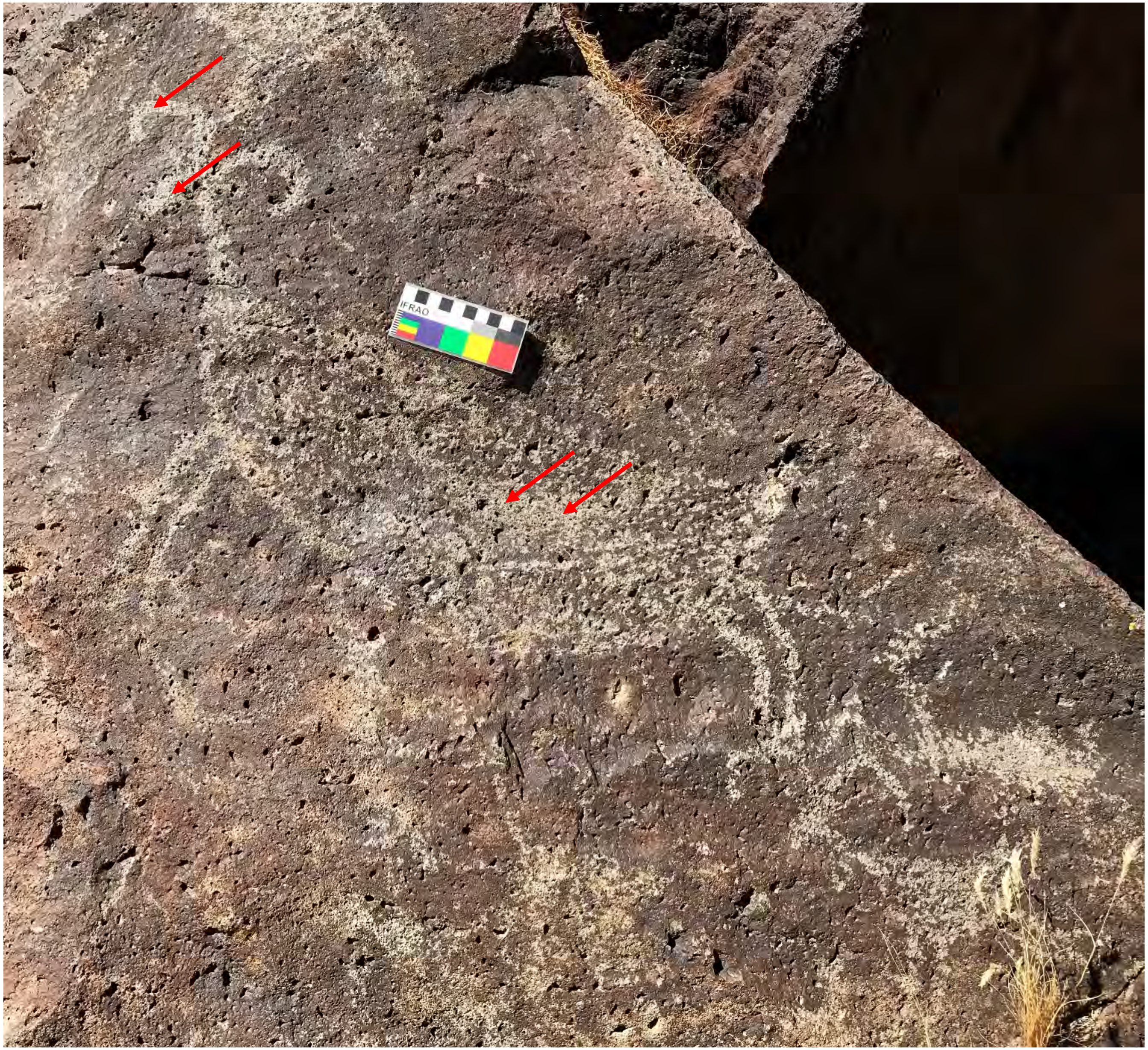

LL7-1

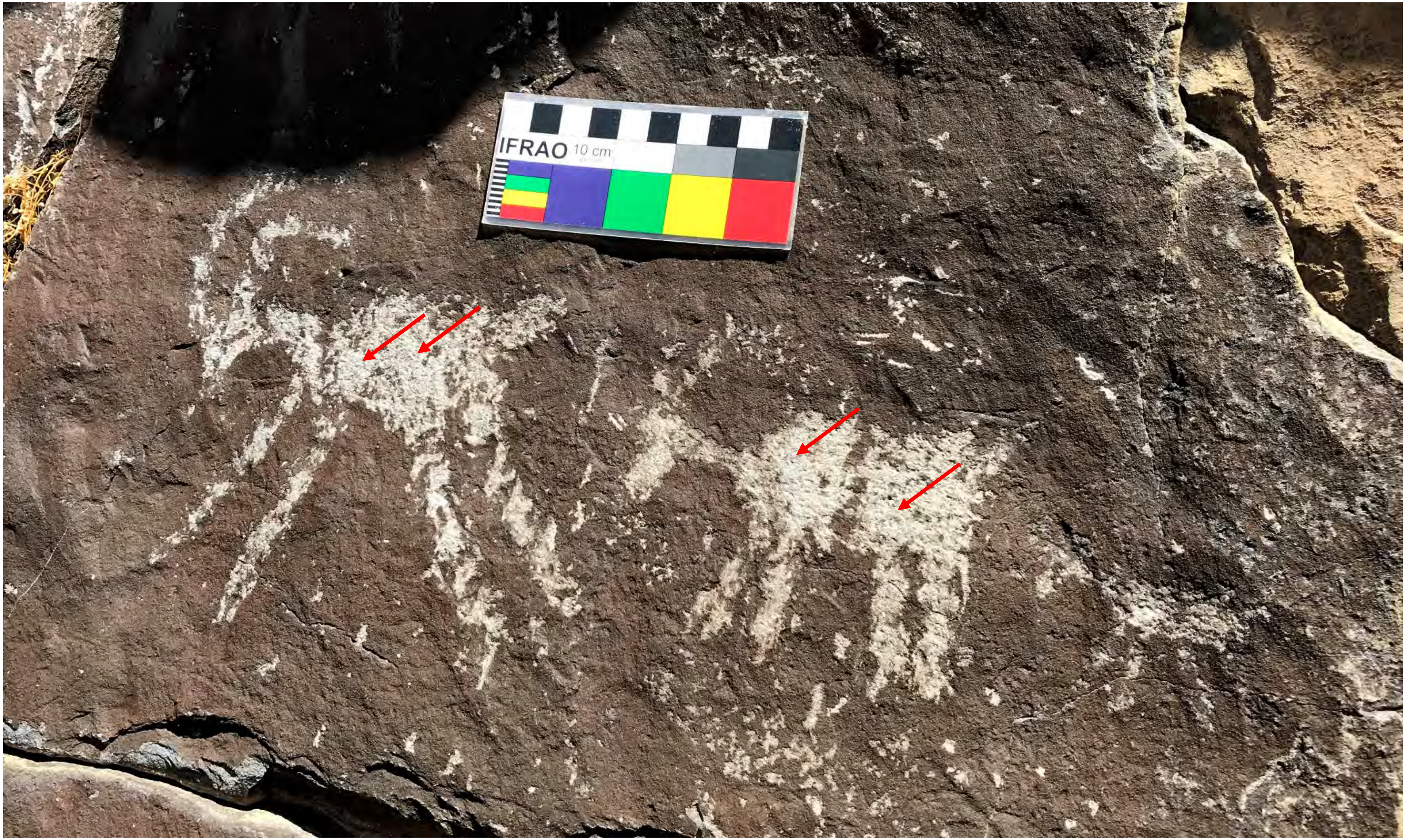

LL7-2

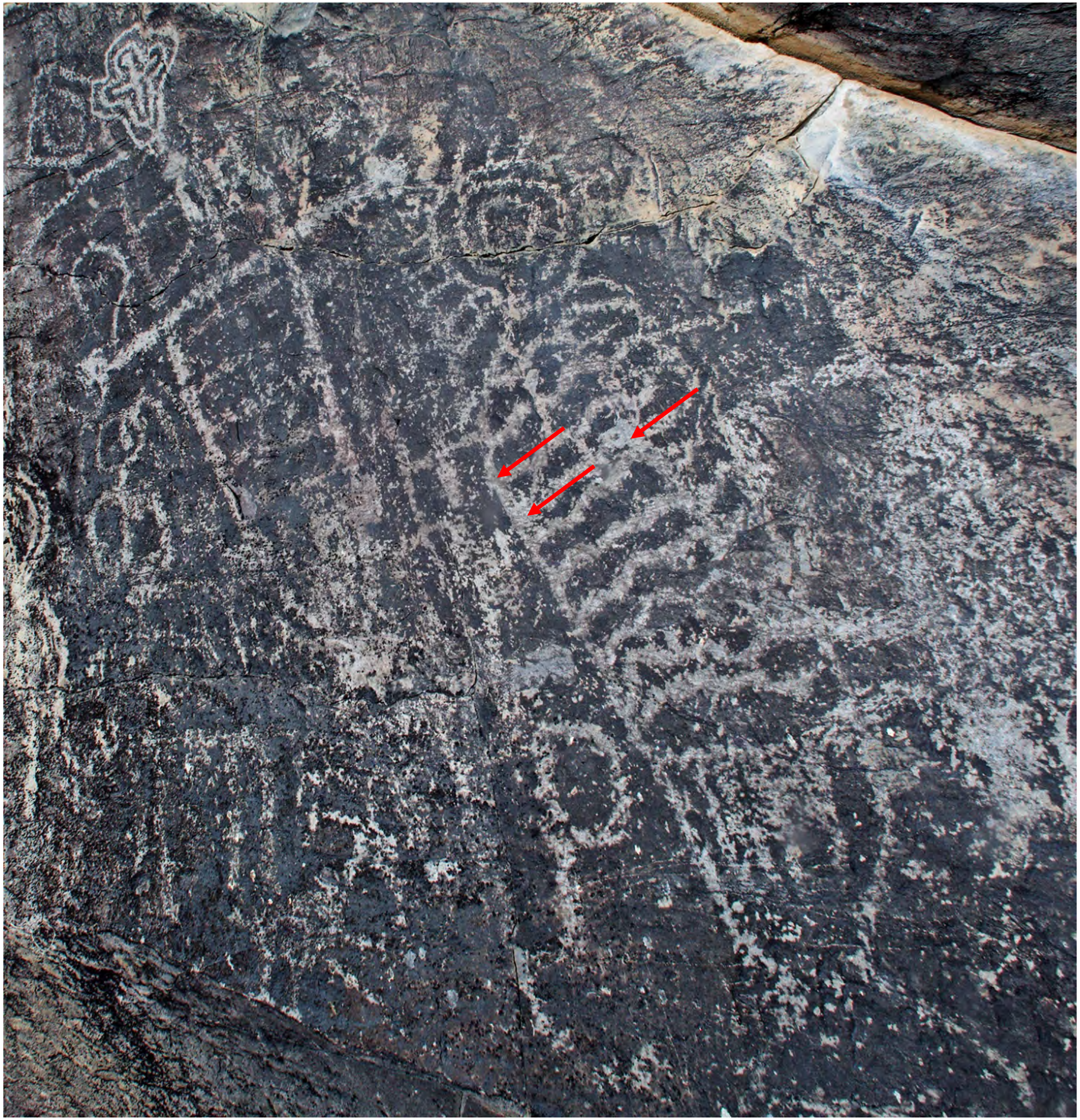

Reproduced under a CC BY license, with permission from Tom Hnatiw, original copyright 2020.

LL7-3

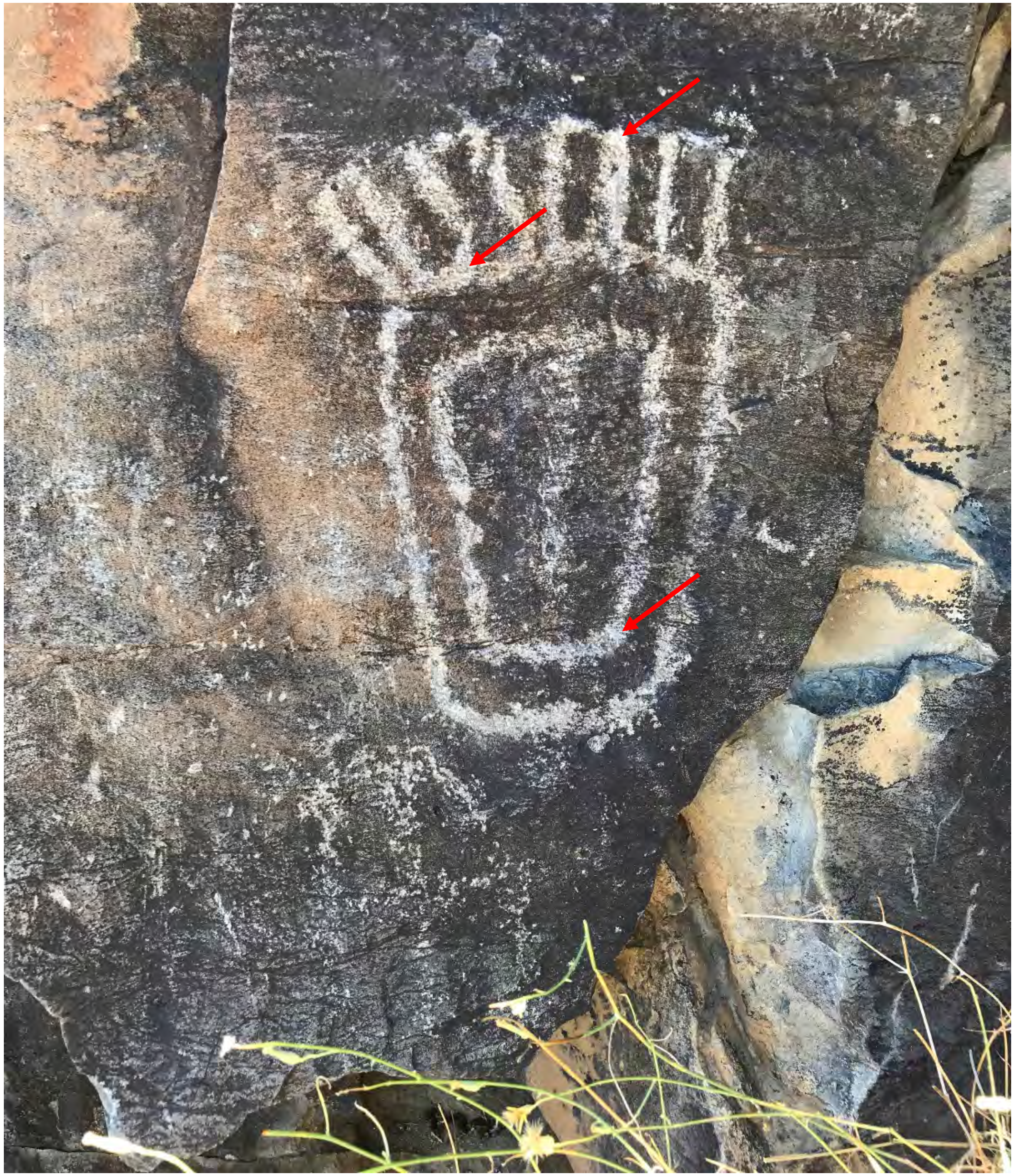

LL7-4

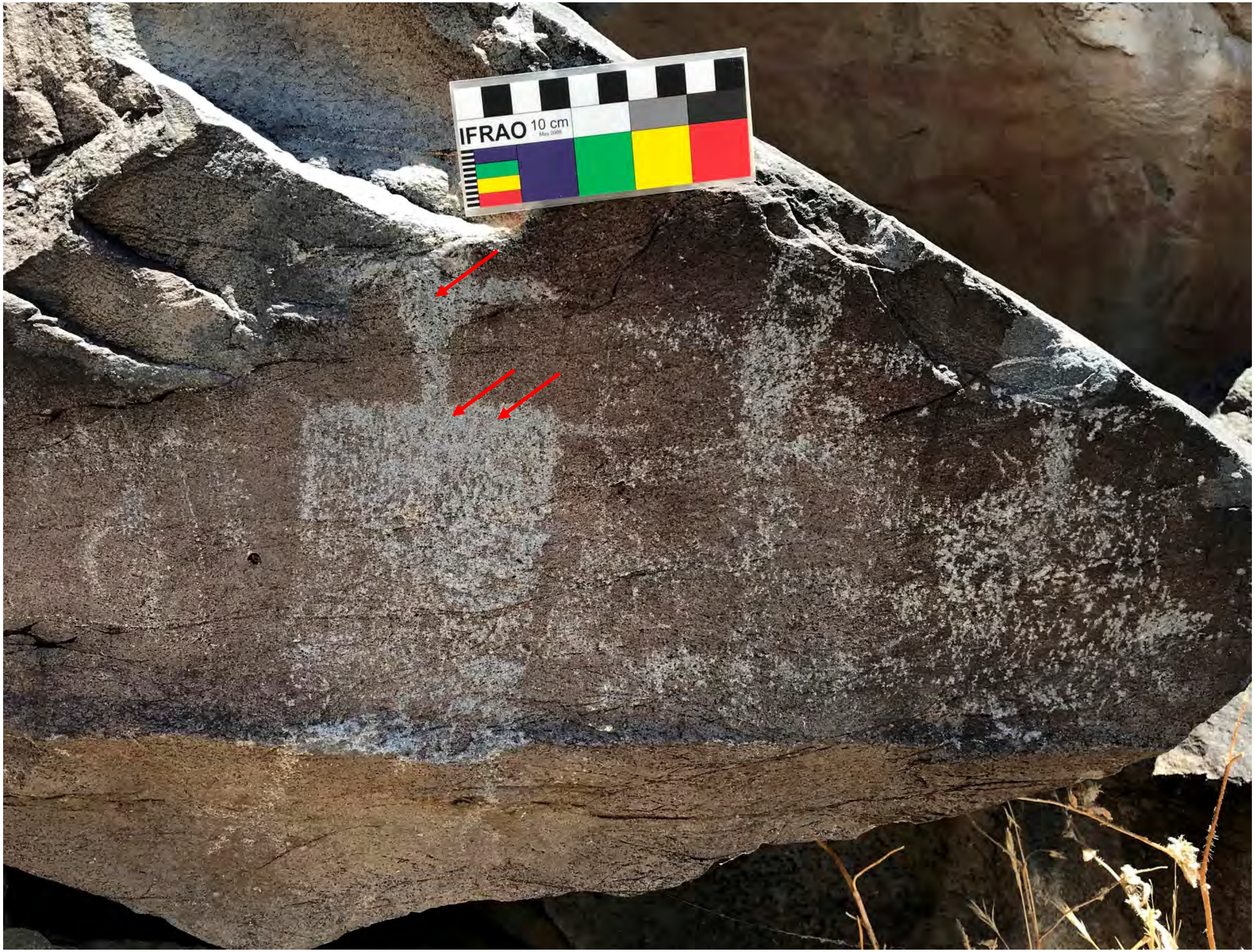

LL4-1 and LL4-2

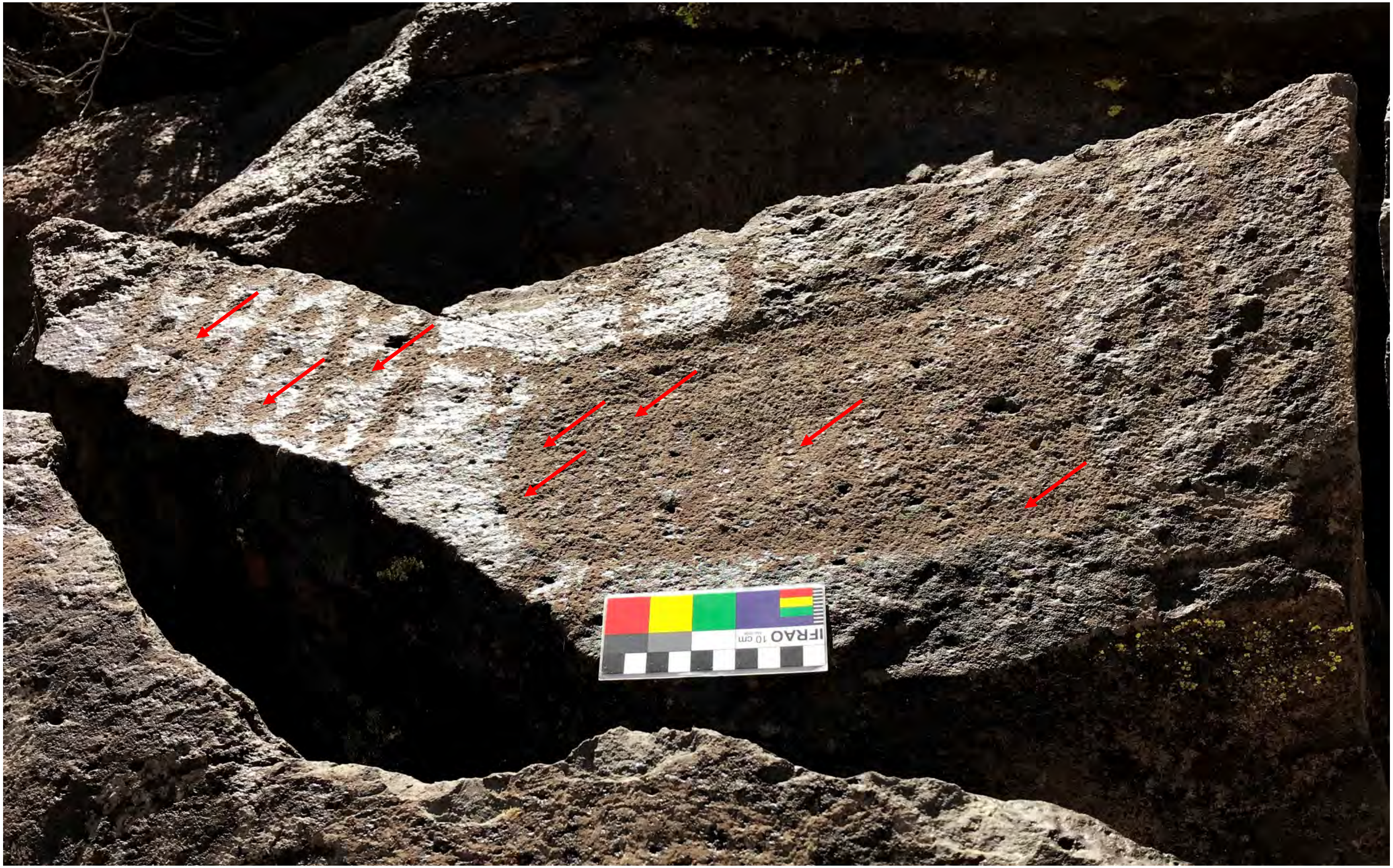

Supplement: S2 Fig — (PDF) [file pone.0235421.s003.pdf]
