# Supplementary material for: Geochemical studies on rock varnish and petroglyphs in the Owens and Rose Valleys, California
Source: PLoS One. 2020 Aug 5;15(8):e0235421. doi: 10.1371/journal.pone.0235421 (PMC7405993; doi:10.1371/journal.pone.0235421)
Supplement: S3 Fig — The x-axis represents successive laser shots on the same spot. Each laser shot corresponds to a depth increment of about 50–100 nm. (PDF) [file pone.0235421.s004.pdf]

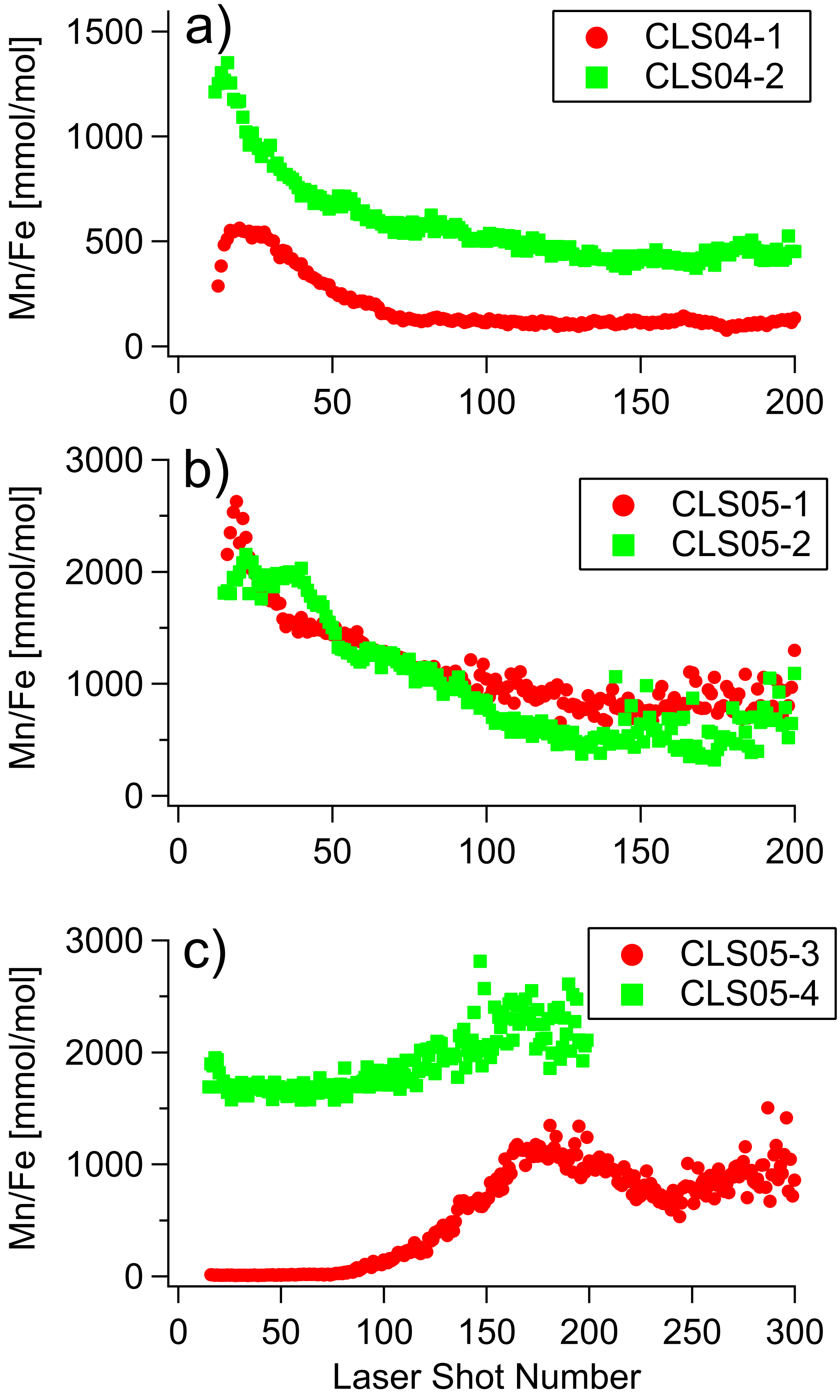

S4 Figure. fs-LA-ICPMS spot measurement profiles on rock varnish samples. The x-axis represents successive laser shots on the same spot. Each laser shot corresponds to a depth increment of about 50-100 nm.
