# Supplementary material for: Geochemical studies on rock varnish and petroglyphs in the Owens and Rose Valleys, California
Source: PLoS One. 2020 Aug 5;15(8):e0235421. doi: 10.1371/journal.pone.0235421 (PMC7405993; doi:10.1371/journal.pone.0235421)
Supplement: S4 Fig — a) without inclination correction, b) with correction using the "attenuated cosine" correction equation (see text). (PDF) [file pone.0235421.s005.pdf]

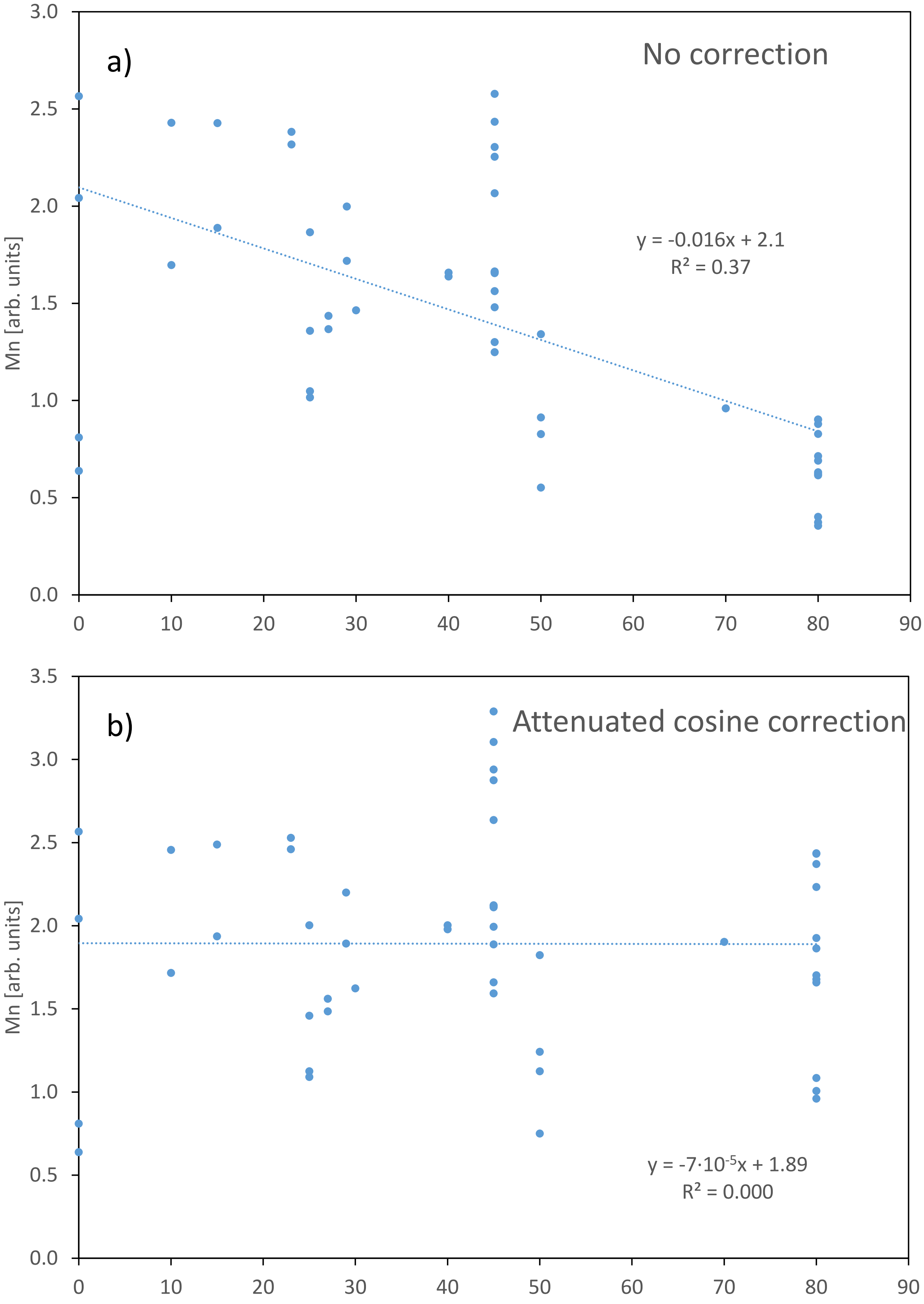

S5 Figure. Mn surface density versus surface inclination. a) without inclination correction, b) with correction using the "attenuated cosine" correction equation (see text).
