# Supplementary material for: Geochemical studies on rock varnish and petroglyphs in the Owens and Rose Valleys, California
Source: PLoS One. 2020 Aug 5;15(8):e0235421. doi: 10.1371/journal.pone.0235421 (PMC7405993; doi:10.1371/journal.pone.0235421)
Supplement: S5 Fig — (PDF) [file pone.0235421.s006.pdf]

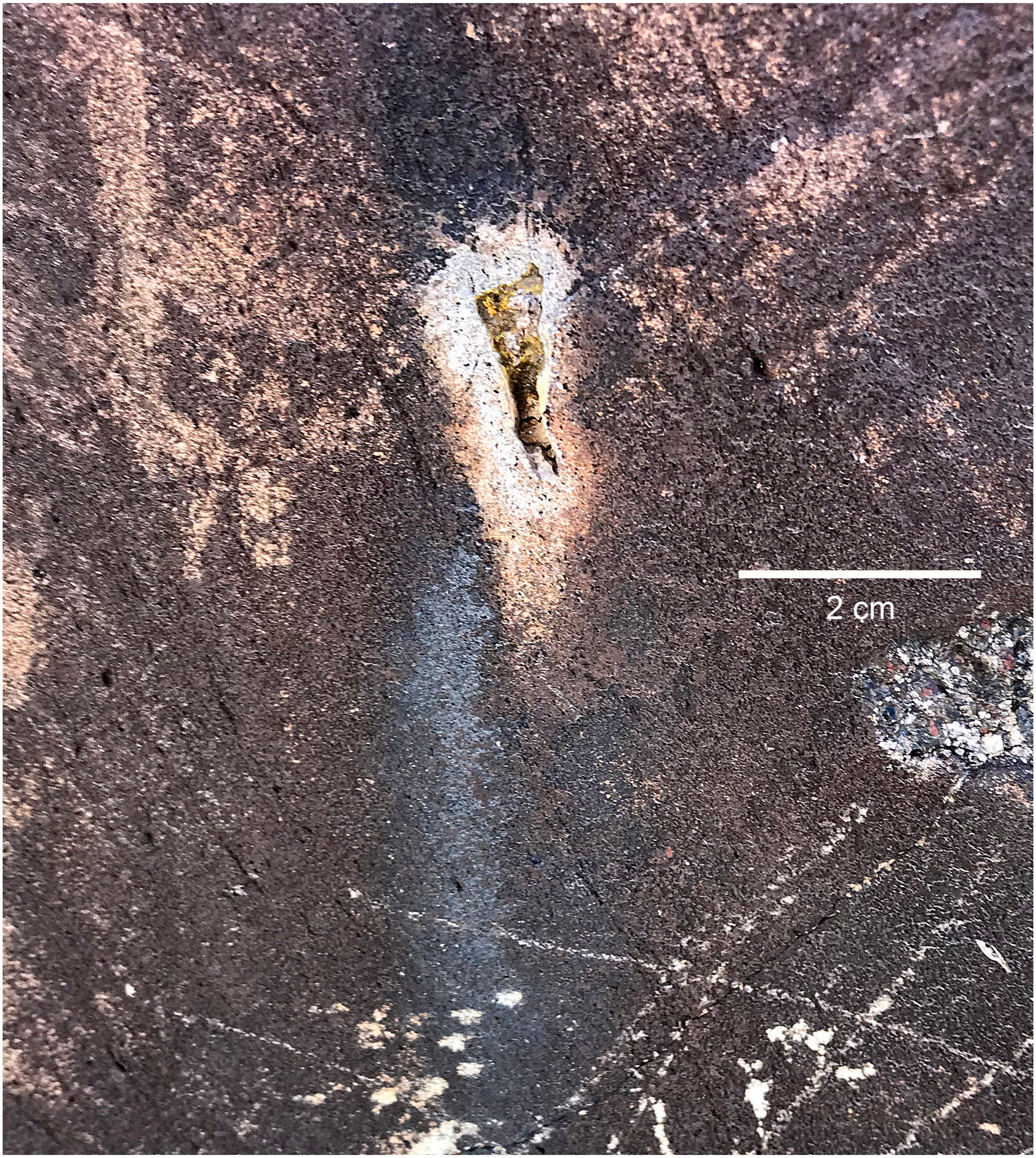

S6 Figure: Image of the varnish around a large olivine phenocryst on a near-vertical rock surface at Little Lake.
