# Supplementary material for: Geochemical studies on rock varnish and petroglyphs in the Owens and Rose Valleys, California
Source: PLoS One. 2020 Aug 5;15(8):e0235421. doi: 10.1371/journal.pone.0235421 (PMC7405993; doi:10.1371/journal.pone.0235421)
Supplement: S6 Fig — Plot of the Mn apparent accumulation rate, RMn, versus surface age, A, of rock varnishes on lava flow surfaces of known age. The error bars represent the standard deviation of the measurements on each lava flow surface. The solid line represents the fit equation, the dotted lines the 95% confidence interval of the fit. (PDF) [file pone.0235421.s007.pdf]

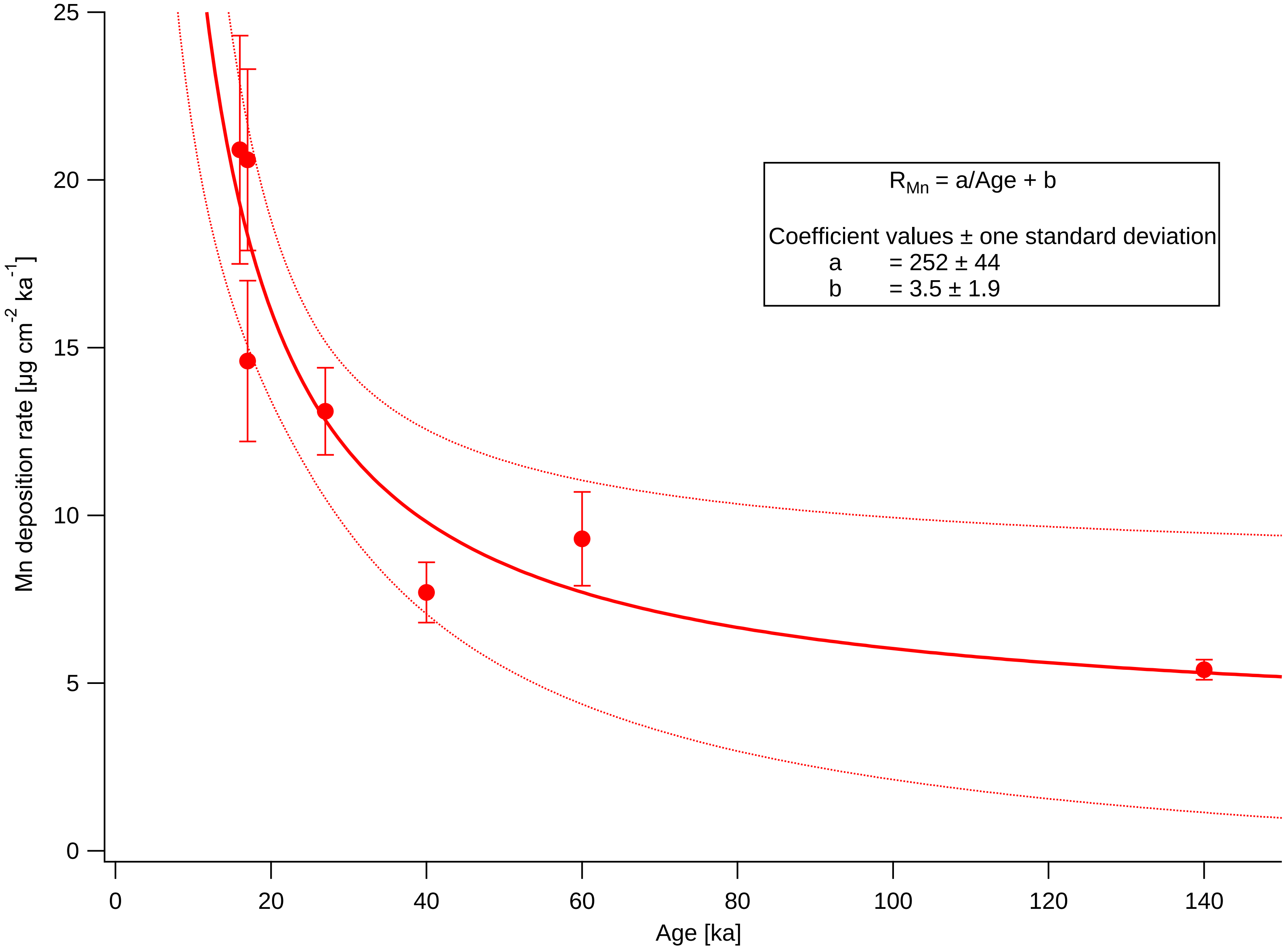

S8 Figure. Mn apparent accumulation rate versus surface age. Plot of the Mn apparent accumulation rate, R_{Mn}, versus surface age, A, of rock varnishes on lava flow surfaces of known age. The error bars represent the standard deviation of the measurements on each lava flow surface. The solid line represents the fit equation, the dotted lines the 95% confidence interval of the fit.
