# Supplementary material for: Geochemical studies on rock varnish and petroglyphs in the Owens and Rose Valleys, California
Source: PLoS One. 2020 Aug 5;15(8):e0235421. doi: 10.1371/journal.pone.0235421 (PMC7405993; doi:10.1371/journal.pone.0235421)
Supplement: S1 Appendix — (PDF) [file pone.0235421.s008.pdf]

**S7 Appendix. Influence of microcolonial fungi on rock varnish at the Conejo Mine site, California.**

This supplement contains material provided to us during the review process by Dr. Ronald I. Dorn. It provides a cautionary note on small-scale variability and influences other than time that can affect varnish areal density.

The figure below shows microscopic cross-sections of varnish from a petroglyph sampled at the Conejo Mine rock art site located about 30 km east of our Little Lake site. The results from age determinations by the cation ratio method (12,000±600 a BP) and the varnish microlamination (VML) method (11,100 a BP) were published associated with the sample code CM15 in the paper by Whitley (2013). The VML work was analyzed by two individuals working separately, Tanzhuo Liu and Ron Dorn.

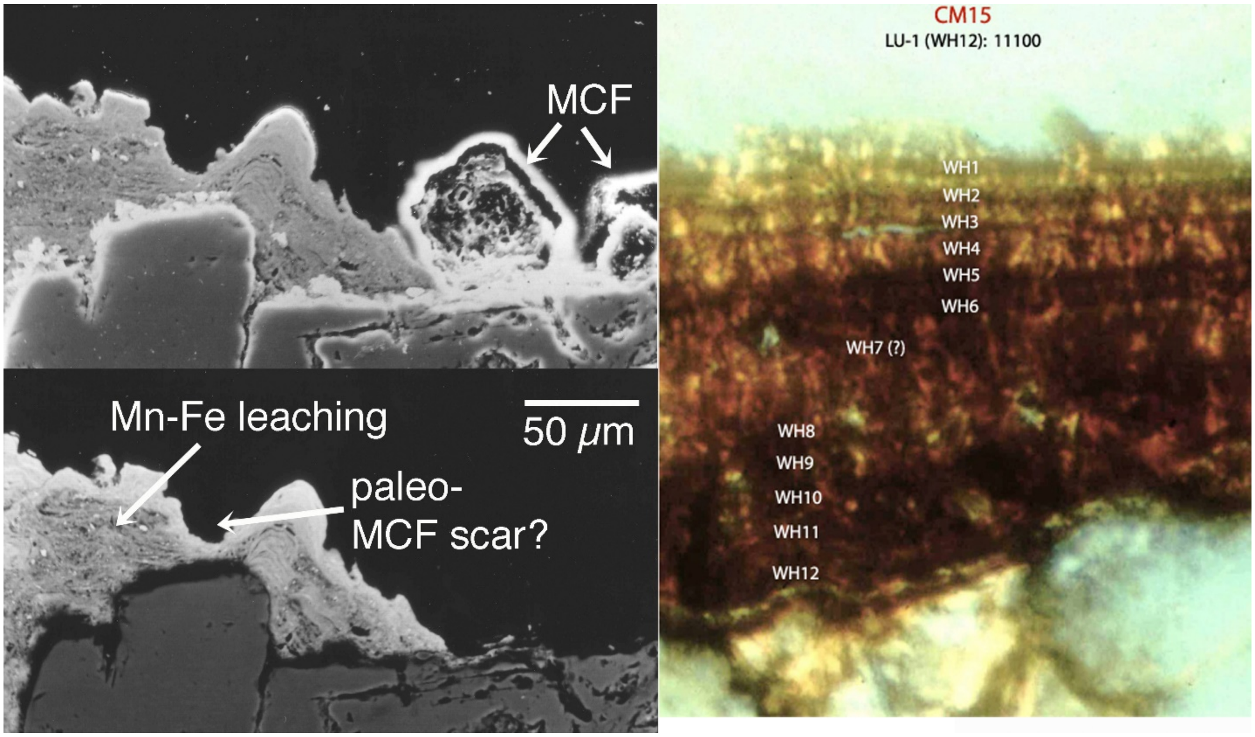


The ultra-thin section, on which the VML dating is based, is shown on the right. This section was one very few among the many sections prepared by Dorn that showed undisturbed varnish and allowed successful dating. The others were covered with microcolonial fungi that dissolve Mn and Fe and mobilize varnish. This means that the total mass of varnish on the petroglyph would not reflect its minimum age, but likely provide an artefactually lower age.

The images on the left are a secondary electron image (upper left), which shows microcolonial fungi (MCF) growing adjacent to the varnish, and a back-scattered electron image (lower left), which reflects the atomic number of the imaged material and shows details of the varnish. Note the areas with voids in the varnish that reflect potential dissolution of the varnish by organic acids, which can leach Mn-Fe oxyhydroxides in variable amounts that are not time dependent.

This petroglyph was in a location where water would accumulate in a sandy wash. The water from a tributary drained a region with relatively impermeable sediment. This meant that runoff would drain to the main wash near this petroglyph. The water would sink into the main wash, and humidity levels are much higher. The net result was an abundance of MCF growing on rock surfaces, including the sampled petroglyph.

This is but one example of a microenvironment that can cause several issues complicating the method that the authors use: (1) relatively fast rate of varnishing because of the relatively moist setting in a dry region; (2) complete removal of varnish by microcolonial fungi; (3) leaching of Mn-Fe from the varnish. The first complication works in opposition to #2 and #3, and there is no way to know what factor influences Mn-accumulation more and how to extract time from the Mn accumulation,

---------------------------------------------------------------

Whitley, D. S., Rock art dating and the peopling of the Americas: Journal of Archaeology, 2013, 15 p., doi:10.1155/2013/713159, 2013.
