# Supplementary material for: Geochemical studies on rock varnish and petroglyphs in the Owens and Rose Valleys, California
Source: PLoS One. 2020 Aug 5;15(8):e0235421. doi: 10.1371/journal.pone.0235421 (PMC7405993; doi:10.1371/journal.pone.0235421)
Supplement: Attachment — Submitted filename: rand_over_age_vs_age.pdf [file pone.0235421.s010.pdf]

	A	B
1	age (ka)	randbetween (0-20)/age
2	1	8
3	10	0.5
4	20	0.85
5	30	0.56666667
6	40	0.1
7	50	0.36
8	60	0.26666667
9	70	0.15714286
10	80	0.175
11	90	0.16666667
12	100	0.04
13	110	0.01818182
14	120	0.06666667
15	130	0.03846154
16	140	0.00714286
17		
18		

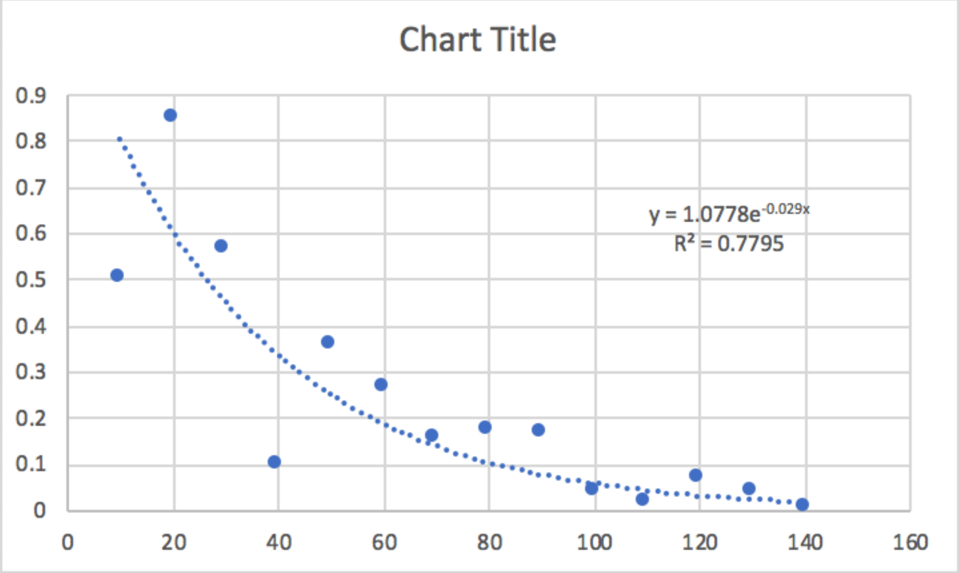

Formula Bar
