# Supplementary material for: Geochemical studies on rock varnish and petroglyphs in the Owens and Rose Valleys, California
Source: PLoS One. 2020 Aug 5;15(8):e0235421. doi: 10.1371/journal.pone.0235421 (PMC7405993; doi:10.1371/journal.pone.0235421)
Supplement: Attachment — Submitted filename: review comments.docx [file pone.0235421.s009.docx]

Review comments: this paper studies rock varnish and petroglyphs in Owens and Rose Valleys, California by XRF and fs-LA-ICP-MS. Detailed geochemical features are given and the authors explained the elemental enrichment patterns. Based on previous archaeological studies, the authors tried to date the relative age of these rock art. The results are reliable and worthy of publication. I think this manuscript can be accepted after moderate revision. The paper should be modified in the two following aspects.

1. This content of this paper is too long and redundant. The language should be brief and to the point, especially for the introduction and methods part. Here I just list some examples that you need to revise. For line 96-120, you do not need to spare so much space to demonstrate what you have done in your previous work. For the geological background part, you do not need write much detail about local vegetation species, climate fluctuation history, human source and occupation history in this region, etc. Most of these information is irrelevant to your work. And please ONLY cite references which are useful to support you point of view.

2. I am not satisfied with the existing sequence of your figures. You first show the raw data, then the correlation curves and last the photograph of rock art. I think it is much better to show the satellite image of sampling site and photos of rock varnish first, and your laboratory analysis should be put last.
