# Supplementary material for: Geochemical studies on rock varnish and petroglyphs in the Owens and Rose Valleys, California
Source: PLoS One. 2020 Aug 5;15(8):e0235421. doi: 10.1371/journal.pone.0235421 (PMC7405993; doi:10.1371/journal.pone.0235421)
Supplement: Attachment — Submitted filename: Response_to_Reviewers.docx [file pone.0235421.s011.docx]

Journal Requirements:

*When submitting your revision, we need you to address these additional requirements.*

*1. Please ensure that your manuscript meets PLOS ONE's style requirements, including those for file naming. The PLOS ONE style templates can be found at* [*https://journals.plos.org/plosone/s/file?id=wjVg/PLOSOne_formatting_sample_main_body.pdf*](https://journals.plos.org/plosone/s/file?id=wjVg/PLOSOne_formatting_sample_main_body.pdf) *and* [*https://journals.plos.org/plosone/s/file?id=ba62/PLOSOne_formatting_sample_title_authors_affiliations.pdf*](https://journals.plos.org/plosone/s/file?id=ba62/PLOSOne_formatting_sample_title_authors_affiliations.pdf)

OK

*2. In your Methods section, please provide additional information regarding the permits you obtained for the work. Please ensure you have included the full name of the authority that approved the field site access and, if no permits were required, a brief statement explaining why.*

We included the following text:

Permission for the measurements at Little Lake was obtained from the owners (Little Lake Duck Club, Inc.). No permit was required for the collection of small rock samples and non-invasive measurements by pXRF on public lands. No samples of cultural heritage material were taken.

*3. We note that the Supporting Information Figures in your submission contain [map/satellite] images which may be copyrighted. All PLOS content is published under the Creative Commons Attribution License (CC BY 4.0), which means that the manuscript, images, and Supporting Information files will be freely available online, and any third party is permitted to access, download, copy, distribute, and use these materials in any way, even commercially, with proper attribution. For these reasons, we cannot publish previously copyrighted maps or satellite images created using proprietary data, such as Google software (Google Maps, Street View, and Earth). For more information, see our copyright guidelines:* [*http://journals.plos.org/plosone/s/licenses-and-copyright*](http://journals.plos.org/plosone/s/licenses-and-copyright)*.*

*We require you to either (1) present written permission from the copyright holder to publish these figures specifically under the CC BY 4.0 license, or (2) remove the figures from your submission:*

*1.    You may seek permission from the original copyright holder of the Supporting Information Figures to publish the content specifically under the CC BY 4.0 license.*

*We recommend that you contact the original copyright holder with the Content Permission Form (*[*http://journals.plos.org/plosone/s/file?id=7c09/content-permission-form.pdf*](http://journals.plos.org/plosone/s/file?id=7c09/content-permission-form.pdf)*) and the following text:*

*“I request permission for the open-access journal PLOS ONE to publish XXX under the Creative Commons Attribution License (CCAL) CC BY 4.0 (*[*http://creativecommons.org/licenses/by/4.0/*](http://creativecommons.org/licenses/by/4.0/)*). Please be aware that this license allows unrestricted use and distribution, even commercially, by third parties. Please reply and provide explicit written permission to publish XXX under a CC BY license and complete the attached form.”*

*Please upload the completed Content Permission Form or other proof of granted permissions as an "Other" file with your submission.*

*In the figure caption of the copyrighted figure, please include the following text: “Reprinted from [ref] under a CC BY license, with permission from [name of publisher], original copyright [original copyright year].”*

We obtained a photograph of the rock art element and copyright permission from the photographer. The form will be uploaded.

*The following resources for replacing copyrighted map figures may be helpful:*

*USGS National Map Viewer (public domain):* [*http://viewer.nationalmap.gov/viewer/*](http://viewer.nationalmap.gov/viewer/)

We produced a new map using the USGS National Map Viewer.

*4. Please include captions for your Supporting Information files at the end of your manuscript, and update any in-text citations to match accordingly. Please see our Supporting Information guidelines for more information:* [*http://journals.plos.org/plosone/s/supporting-information*](about:blank)*.*

OK.

***Reviewer #1:***

*Review comments: this paper studies rock varnish and petroglyphs in Owens and Rose Valleys, California by XRF and fs-LA-ICP-MS. Detailed geochemical features are given and the authors explained the elemental enrichment patterns. Based on previous archaeological studies, the authors tried to date the relative age of these rock art. The results are reliable and worthy of publication. I think this manuscript can be accepted after moderate revision. The paper should be modified in the two following aspects.*

We thank the reviewer for his comments. The reviewer’s remarks are in italics, our responses in regular font.

*1. This content of this paper is too long and redundant. The language should be brief and to the point, especially for the introduction and methods part. Here I just list some examples that you need to revise. For line 96-120, you do not need to spare so much space to demonstrate what you have done in your previous work. For the geological background part, you do not need write much detail about local vegetation species, climate fluctuation history, human source and occupation history in this region, etc. Most of these information is irrelevant to your work. And please ONLY cite references which are useful to support you point of view.*

In our writing, we strove to include all essential information. In previous papers on related subject matter, we were criticized by reviewers for not providing enough background information. Different reviewers tend to have different tastes on how detailed and comprehensive introductions and background discussions should be. We refer to reviewer 2, who writes: “The writing is excellent. The presentation of methods and results is clear. The literature analyzed is appropriate and balanced.” Given that this is a highly interdisciplinary paper, we cannot assume that all readers are familiar with Southwestern archaeology and prehistory, geochemistry, climate, and ecology. Yet all these dimensions are crucial to understanding varnish formation (a biogeochemical process dependent on geology, climate, microbes, etc.) and petroglyph creation. The history of climate fluctuations affects varnish growth as well as human behavior relevant to the time of rock art creation, which is a central issue here. We did remove a long sentence in the paragraph line 96-120, which made a similar statement as made in the conclusions.

*2. I am not satisfied with the existing sequence of your figures. You first show the raw data, then the correlation curves and last the photograph of rock art. I think it is much better to show the satellite image of sampling site and photos of rock varnish first, and your laboratory analysis should be put last.*

The map of the sampling sites is actually the first figure referenced (although it is in the Supplement as S2). At the editor’s discretion, we are willing to move it into the main paper. The supplement figure with the photographs (S3) was also pulled forward. We moved the figure with the rock art photos (new Fig. 4) to the beginning of the section dealing with petroglyphs.

***Reviewer #2:***

*Rates of varnish accretion are highly dependent on microenvironment. Sometimes, the word microenvironment is misinterpreted to mean aspect or the direction an outcrop faces. Examination of south-facing versus north-facing aspects in a particularly dry environment could very well reveal no significant differences in accretion rates. In more mesic settings, however, aspect can play a major role. So can other forcing factors on microclimate such as cold-air drainage, subtle rainshadow effects, major rainshadow effects, inversions, the thermal conductivity of the host rock material, proximity to soil moisture such as along a wash that would have collected runoff. This small list only begins to account for variability in moisture availability on rock surfaces.*

We couldn’t agree more and emphasize variability at numerous places in the paper. In contrast to most previous authors, we also provide a quantitative statistical analysis of variability. We have added the factors mentioned by the reviewer to the table of factors affecting varnish growth, which we include as a supplemental table (see below). The following sentence was inserted in the text concerning the effect of cardinal orientation: “It is, however, possible that in more mesic settings, especially with rain or dew coming from a preferred direction, cardinal orientation may play a significant role.”

*The impact of microenvironment has been noted in the only known method to measure both varnish thickness and varnish age, varnish microlaminations. The authors are very familiar with the papers of Tanzhou Liu and co-authors. Certainly Dr. Liu is the world’s foremost authority on the issue.

David Whitley’s seminar paper on the use of VML in the dating of petroglyphs in the southwest (Whitley, 2013) involved blind testing of Dr. Tanzhuo Liu and myself (Ron Dorn). We analyzed each other’s ultra-thin cross-sections and Dr. Whitley compiled the findings with his world-renowned expertise for rock art. The result was synthesized and condensed down. What was not apparent in the paper but was apparent to all of us (Dave, Tanzhuo and myself) was the highly variable thicknesses of varnishes with similar ages – and this is due to the effect of microclimate.

My own research on VML and its use certainly validates Dr. Liu’s findings. I used VML to estimate the ages of debris flows in metropolitan Phoenix (Dorn, 2010) and noted this problem, and yet the VML method was still able to predict a historical debris flow event that occurred four years later (Dorn, 2016). In other research project, variable rates of varnishing was also noted to be highly dependent on microenvironment (Dorn, 2014).

However, there are other papers taking different approaches to understanding varnishing that indicate the same thing: that microenvironment greatly influences varnish rates of accretion. The lead accumulation research of Spilde and colleagues was made possible because the more mesic varnishes they analyzed had rates of accretion faster than those found in more zeric settings (Spilde et al., 2013). A critique of the cation-ratio dating method notes a similar problem (Krinsley et al., 1990).

In an earlier paper on the use of XRF and Mn abundance to estimate petroglyph ages from Saudi Arabia, I recommended the authors put together a table of complications that could interfere with the use of their technique in estimating age. I was very pleased with the table they produced (Macholdt et al., 2019), and I suggest that they reproduce this table here in this paper. Some of the factors that they analyze could be in play, and the readers would benefit from their detailed analysis.*

We fully agree with the reviewer on the importance of microenvironmental variables on varnish accretion and have pointed this out at several places in our paper. We appreciate his suggestion of including an updated version of our table from Macholdt et al. (2019). We thought about doing this when preparing the original version of the paper, but decided against it as we did not want to repeat material that has already been published and we also didn’t want to make this already long paper even longer. Reviewer 1 criticized already us for discussing too much our earlier work. As a meaningful compromise, we now include an updated version of the Table as a supplement.

However, our use of the normalized density, N_Mn_, mitigates much of this variability, since for age estimation we do not look at the absolute amount of Mn on the rock art, but at the amount relative to an adjacent intact spot, which has been exposed to the same microenvironmental conditions.

*I also offer for the author’s use and give my permission for its use in this paper, an illustration that relates to the research presented by Whitley (2013). One of the petroglyphs analyzed in his Table 1 is CM15 an “X-motif”. The original publication of the cation-ratio age of 12000±600 yrs cal BP was not too dissimilar to the VML age confirmed by both myself and Tanzhul Liu of ca. 11,100 yrs cal BP — keeping in mind that both are minimum ages for the underlying engraving. What this one result in the table does not convey involved the reality that this petroglyph would not have shown much Mn-accumulation with the XRF approach.

Please examine the illustration that I attach in high resolution. One of several VML ultra-thin sections is shown on the right. To obtain these ultra-thin section was a real pain! Almost all of the tiny samples from the petroglyph were covered with microcolonial fungi that dissolve Mn and Fe and mobilize varnish. This means that the total mass of varnish in no way reflects its minimum age. The process to find VML sequences that were not disturbed by MCF was laborious and painstaking.

Note: the left images are secondary electrons (upper left) that show the microcolonial fungi, and back-scattered electrons (lower left) that show atomic number. Please note the areas with voids in the varnish that reflect another issue entirely … that even if the MCF do not dissolve the varnish completely, the organic acids (my assumption) can still leach Mn-Fe in variable amounts that are not time dependent.

Why did I highlight this particular petroglyph and these images? This petroglyph was in a location where water would accumulate in a sandy wash. The water from a tributary drained a region with relatively impermeable sediment. This meant that runoff would drain to the main wash near this petroglyph. The water would sink into the main wash, and humidity levels are much higher. The net result was an abundance of MCF growing on rock surfaces, including the sampled petroglyph.

This is but one example of a microenvironment that can cause several issues complicating the method that the authors use: (1) relatively fast rate of varnishing because of the relatively moist setting in a dry region; (2) complete removal of varnish by microcolonial fungi; (3) leaching of Mn-Fe from the varnish. The first complication works in opposition to #2 and #3, and there is no way to know what factor influences Mn-accumulation more and how to extract time from the Mn accumulation

Why was this petroglyph sampled if it would be such a pain? It was because Dr. Whitley thought it was particularly critical and asked that the effort be made to find a suitable sample.

Thus, you can see that after years of making thin sections and examining cross-sections, I am incredibly skeptical that any method of dating petroglyphs involving the accumulation of manganese can yield reliable results.*

In response to the reviewer’s comment, we have crafted in collaboration with him a supplement showing his figure and discussing its implications. We refer the reader to this supplement at the end of the section on variability of varnish areal density.

*WHY DID I RECOMMEND ACCEPTANCE WITH MINOR REVISIONS IF I AM SO SKEPTICAL? The reason is that the authors did an outstanding job of presenting their research. They are clearly excellent scientists and I think the research needs to be published. The writing is excellent. The presentation of methods and results is clear. The literature analyzed is appropriate and balanced.

WHY DID I RECOMMEND MINOR REVISIONS? I would urge the authors to consider adding a table similar to the one they presented in Macholdt et al. (2019). I am sure that their thinking on potential complications with their method have evolved since they presented their outstanding table. The table could be modified to deal with the realities of
working with petroglyphs and study sites in the southwestern Great Basin of the USA.
I do not feel like it is my place to suggest that publication be conditional on the presentation of this cautionary table. I respect the authors and would trust their judgement given the perspective I am providing as a reviewer with substantial experience in this field area.
I also do not feel like it is my place even urge the authors to include the illustration I provide as an example of a petroglyph that would be highly problematic to analyze with their technique. Again, the authors fully understand the different approaches ... their approach goes for a much larger surface area. The one that I have found to be most
reliable and robust tries to find the best sampling area.

I appreciate the opportunity to provide the authors this feedback.*

We thank the reviewer for his comments.

***Reviewer #3:***

*Meinart et al., set out to test whether they can date petroglyphs in Owens and Rose valleys in the western US based on the re-varnishing rates of the petroglyphs. They produced a calibration curve based on pXRD and IC-PMS measurements of varnish formed on basalt of known ages. In the end, they don’t use the calibration curve from the western US to derive the re-varnishing rate but one from Saudi Arabia that they previously calculated.

Firstly, I applaud the work Prof. Meinart and his students have been doing in the field of rock varnish research. However, there are a few issues in this work that must be addressed before this paper can be published.

Major
1. It took me a while to understand what exactly was done because of the terms “areal density” and “deposition rate”, which are a bit confusing. I suggest using the term “Lateral varnish growth rate”. In addition, it’s not deposition rate (1/depth or 1/m) but lateral coverage rate (1/area or 1/m^2) – how fast does varnish cover the rocks’ surface.*

Unfortunately, the reviewer misunderstood our terminology. We do mean, in fact, areal density. A definition can be found in Wikipedia, among many other sources: “The area density (also known as areal density, surface density, superficial density, areic density, mass thickness, column density, or density thickness) of a two-dimensional object is calculated as the mass per unit area. The SI derived unit is: kilogram per square metre (kg·m−2).” (<https://en.wikipedia.org/wiki/Area_density>). We have used this term already in several peer-reviewed articles, and no reviewer has previously found any problem with its use. To be totally explicit, we included the definition of areal density on its first use in the introduction.

The same applies to “deposition rate”. According to the generally accepted conceptual model of varnish formation, it is a sedimentary deposit or accretion (as also stated by the reviewer below). Thus the time derivative of areal density (mass area^-1^) is a deposition or accumulation rate (vertical, not lateral!) and has units of (mass area^-1^ time^-1^). Again, all previous reviewers of our work found no problem with this. We changed the term “deposition rate” to “accumulation rate” for a more neutral expression, and also included its definition in the introduction.

*2. The question you are asking is how fast does varnish spread laterally. The most straight forward way to do this is by light image processing (as was previously done by Bednarik).*

Straightforward, but problematic. Iron oxyhydroxides also cause a color change, but we have definitely shown in this and our previous papers that Fe accumulation does not correlate with age. Fungal growth can also produce rock darkening, as we have found at several locations.

*The choice to use a pXRF complicates the ability to calculate the parameter you are looking for, because [Mn] is a function of two things: Is there varnish and what is the [Mn] concentration of the varnish (this in itself a function of vertical sedimentation rate and the climatic condition that the varnish formed in).*

The choice of Mn actually eliminates any ambivalence, as the presence of Mn oxyhydroxides defines rock varnish. So, only if Mn is present, is there rock varnish. Furthermore, we don’t determine “[Mn]”, which would be the concentration of Mn (presumably in the varnish), as was done for instance by JC Bard, and which was not very successful. Rather, we measure Mn areal density, which tell us how much Mn, in the form of varnish, is present on the surface.

*In addition, as varnish is a sedimentary accretion, it first grows in micro-basins and once these are full it starts spreading laterally, so lateral varnish coverage should also be a function of surface roughness. So you have three unknowns and only one measurement, which makes this an underdetermined problem. To try and illustrate my problem, it’s like using the lateral spread of quaternary sediments on a landscape to say something about the age of the sediments.*

We disagree here. There is no evidence that we have ever seen that varnish grows first in microbasins and then spreads laterally. We accept that varnish grows fastest and thickest in microbasins, but it definitely grows all over the rock surface. In fact, varnish grows very well on vertical surfaces that have no microbasins at all. The point of our approach is to make enough measurements to obtain an area-average areal density and accumulation rate, which includes any microbasins, if present.

*3. Your dating is based on the existence of a correlation of [Mn]/m^2/age vs. age (Fig. 4, lines 588-618).*

This is a misunderstanding. Our dating (we actually refrain from claiming that we are “dating”, but only state we “examine the potential of dating”) is based not on a relationship of “[Mn]/m^2/age vs age”, but on areal density vs. age.

*I’m not sure why you are dividing the lateral accumulation by the age and then plotting against age, this by construction would give you an exponential fit every time, with an r^2 value greater than 0.5. For example, I made a simple plot in excel, where column A is age and B is random numbers between (0-20) divided by age. You can see that you can get very high correlations generated in a random way (I chose a high r^2 value case). Because the age and 1/age have a perfect correlation with each other, and you divide this by a random number, on average you should get an r^2 value of at least 0.5. If the original data is slightly correlated with age (say r^2 =0.25) the combined r^2 value would be 0.75. This still means that the data and age are only correlated to 0.25, not 0.75..
This is why Liu and Broecker didn’t get a correlation and when you divided by age you did get one.
I suggest, plotting the lateral deposition (ng/m^2) against age, this is the metric you are interested in. How much varnish formed vs. time. Not how much varnish formed per time vs. time.*

Here, we agree with the reviewer. We were examining whether the “apparent accumulation rate”, i.e., the areal density divided by the age, is constant or decreases with age. A constant apparent accumulation rate would make dating straightforward. In Fig. 3, this would result in a horizontal line. Obviously, the data do not support this, indicating that the growth rate is not constant.

A plot of Mn density vs. age at constant accumulation rate would be a straight line with a positive slope, the accumulation rate. By definition, any such plot would have to go through the origin, since at the moment that the surface is first exposed, there is no varnish. In our new Fig. 3, we show the plot suggested by the reviewer, i.e., areal density vs. age. There is still a strong correlation using a logarithmic fit function. There would also be a good correlation using a linear fit, but the fit would cross the y-axis far above the origin, which violates the condition of no varnish at age zero. The logarithmic fit function includes the origin within its uncertainty, but is so poorly constrained in the age range relevant here, that it cannot serve as a basis for dating.

Our original plot is essentially the first derivative of the new plot, and therefore has the advantage of highlighting the change in accumulation rate with age. It also uses the variable that one would need to use for dating, the accumulation rate, and we feel that it is important to show that this is not a constant. But, as the reviewer points out, this is statistically not robust, and we have therefore changed the discussion in the main text to emphasize that density is not a linear function of age, and moved the plots with accumulation rate vs age with appropriate caveats to the supplement. The text was changed to reflect these points.

By the way, Liu and Broecker’s data plotted as density vs. age actually also yield a weak, but significant correlation (r^2^ = 0.18), but the data are too noisy to provide a meaningful regression. In part, this is likely due to their very selective sampling in the form of making measurements on microbasins, whereas we obtain averages over large surfaces. Since it did not contribute significantly to our discussion, we have removed the plot with Liu and Broecker’s data.

*4. sampling strategy. For this method to be valid, you had to sample the basalt in a random way (otherwise your results are biased, say if you chose varnished areas over none varnished areas). From the methods it is unclear if this is the case. An example would be to draw a 3m line on the surface and measure every 10cm. Was a random method used? This needs to be clarified.*

On a block lava flow, it is impossible to make measurements in the way suggested. In fact, it is not even possible to walk in a straight line because of the extreme unevenness of the surface. Measurements were made by moving across the flow in more or less the same direction, and finding surfaces every few meters that were smooth enough to allow use of the pXRF, while also sampling a range of directions and inclinations. Since it is impossible to visually estimate varnish density on the black basalt, a selection bias is precluded. This information was added to the methods section. *5. Do the concentrations of the major elements from pXRF correlate with major elements from the ICP-MS? Could this be used to further the ability to understand some of the scatter in the data?*

The major element composition in the pXRF is completely dominated by the basalt, whereas in the ICP-MS we only analyzed the varnish. Thus they do not show any relationship.

*6. The petroglyph age assessment. After the Owens data was rejected, the authors turn to use the Saudi data. But this data is 3-4 times slower than what is found in Owens (Lines 405-407). In addition, the age calculation used is linear (10% per 1ka). But the data (Figure 4) shows an exponential growth rate. Given the detailed description of the data, I’m not sure how the Saudi data can be used without a much more elaborate discussion.*

In fact, we are not using the Saudi data, but we are making the hypothetical assumption that the revarnishing rate is linear at 10 %, as clearly stated in the text, and the examine what “implied ages” one gets under these assumptions. We parenthetically state that this is similar to the Saudi rates of 10 -12 %, but this is of no great relevance. The main basis for our choice of hypothesis is that the highest degree of varnish regrowth on the petroglyphs is about 100 %, and that the highest plausible date for the oldest petroglyphs is about 10 ka. The text states: *“The “implied” age scale on the right was chosen by making the hypothetical assumption that full revarnishing occurs in 10 ka, corresponding to a linear revarnishing rate of 10% ka^-1^, in analogy to the revarnishing rates of 10-12% ka^-1^ we had found in Arabia. In the following we examine whether the observed N_Mn_ are consistent with the ages implied under this hypothesis.”* We don’t see how we could state our approach any more clearly.

We then examine what ages are implied for the petroglyphs based on these assumptions, or variations of the assumed rate. Changing the rate by a factor of two clearly gives “implied ages” in conflict with the archaeological information. On the other hand, the chosen assumptions are roughly consistent with the independent estimates from archaeology. This is as far as one can go with the present results, which basically are a feasibility study. We emphasize more than once in the paper that to make further progress, one needs calibration surfaces in the same range of ages as the rock art.

*Minor

Line 69 and 94. This processes is well described in Liu and Dorn (1996) – I recommend citing it.*

Done.

*Line 218. Please add how deep does the pXRF penetrate (if at all)? How does it deal with rough surfaces?*

The following text was added to the methods section.

“The measurement depth of the pXRF is dependent on the energy of the excitation and fluorescence photons, as well as on the composition (atomic number) of the analyte. The software in the instrument takes these factors into account when calculating the results. For the elements considered here, the measurement depth is of the order of a few tens to hundreds of microns.”

Surface roughness increases measurement error. We always measured on as flat and smooth surfaces as possible. Much of this influence is compensated in the case of petroglyphs by the normalization to surrounding intact varnish.

*Line 229. Why are the measurements on the basalt valid and the petroglyphs need to be corrected?*

This is briefly described in the text, and discussed in detail in the cited reference, which we did not want to repeat. Actually, we say: “While the measurement results from the **bare** basalt are valid as provided by the instrument in mass concentration units, the measurements on the **rock varnishes** were **converted** into areal density values, …”. This is because the calibration of the pXRF is for measuring the composition of the rock volume in which the X-ray fluorescence occurs. This is fine for measuring the composition of the host rock basalt (or any rock). For measuring the amount of varnish, we had to obtain a different calibration curve, which relates the amount of Mn in the varnish coating to the X-ray intensity measured by the instrument. This is discussed in detail in Macholdt et al. (2017).

In addition, one has to make a **correction**, because some of the Mn or Fe X-rays come from the basalt, not the varnish. So this has to be subtracted. This correction is very small for Mn, but significant for Fe, explaining the greater error for the latter element. In the text: “To **correct** for the underlying basalt element contribution, the Mn concentration of the unvarnished basalt was determined by conducting a measurement on a nearby freshly exposed rock surface and this value was subtracted from that measured on the varnished surface.”

*Line 411. You use a very different method than Broecker and Liu’s 2001 work. They use very carefully chosen varnishes that display distinct features and then measure the average [Mn] of the downward profile of the varnish and compare that to rainfall amount. You are measuring the surface varnish in an 8mm diameter. I’m not saying Broecker and Liu are right, just that I think the difference in what you are measuring makes this statement problematic.*

We are actually not just measuring the surface, but the integrated amount throughout the varnish. The point we are making here is that Broecker and Liu only see a very weak correlation at the dry end of their data range, so that even from their data one would not expect to see a noticeable dependence. If one looks at their data in the rainfall range < 150 mm, there is no relationship either. This is fully in agreement with our observation of a lack of such a relationship in our data.

*Line 449-458. Would differences in the amount of Fe in the dust make a difference on the Mn/Fe in the varnish?*

I don’t think this has been investigated by anyone so far. Considering the mechanism of varnish formation, I don’t think this is very likely, because there is always lots of Fe in dust relative to Mn, and the enrichment is due to the different physicochemical behavior of the two elements. Also, the Mn/Fe ratio in dust tends not to vary a lot and is usually close to the crustal average.

*Line 499. Please move this sentence to the methods and elaborate on what exactly was done. How many spot measurements are used to characterize one surface?*

We have left the sentence there, because it is needed to inform the reader about how we addressed variability. But we added the following text in the methods: “The measurements were typically made on several spots inside and adjacent to petroglyphs. For each spot, three to five replicate measurements were made by moving the pXRF a few mm or cm (depending on the size of the feature) within or near the petroglyph. Depending on the size and complexity of a given rock art element, one to five such spots were measured on each element. The locations of the spots are marked by arrows in Fig. S3.”

*Line 531-535. These lines must be moved to the methods.*

We added “Six to 18 measurements were made on each lava flow…” in the Methods, but left the text mentioned by the reviewer at its location, because it is needed there to explain the reason for the higher variance of Fe and the basis for calculating standard errors, which are discussed here.

*Line 550: How did you chose the surfaces adjacent to the petroglyphs for the comparison analysis? Are they of the same roughness?*

They were chosen to be as close as possible to the petroglyph measurement spots, and to be as similar as possible in surface characteristics. Text to that extent was added in the methods.

*Line 553-554: When measuring on a small scale you are improving the precision, but your accuracy might be off, i.e. it’s unclear if these few centimeters are representative of the larger population.*

We did what was logistically possible. But given that we measured on multiple nested scales, from mm to hundred km, we feel that our measurements are quite representative. On each lava flow, the measurements only cover a scale of the order of meters to tens of meters, yet the results from the four flows, km apart, are quite similar (250 – 350 µg cm^-2^). On the rock art, the scale is defined by the size of the rock art, and here we use replication at each spot for the smallest scale, and replication at the petroglyph scale by measuring on multiple spots.

*Fig 3. What do the error bars represent?*

The error bars represent the standard deviation of the measurements on each lava flow surface. This information was added to the caption.

*Line 612. remove comma after surfaces*

I don’t understand why. This comma precedes a list of items, and would thus be correct. But to make things clearer, we added “i.e.,”.

*Line 702. Fig. 4. What are the vertical error bars? In light of the discussion of lines 676-701 and the assignment of 33% uncertainty for the Saudi Arabia data (line 110) they seem very small to me. How did you aggregate the error?*

The error bars represent the standard deviations of replicate measurements on each rock art element (as given in Table 6). This information was added to the caption. It would not be appropriate to try and include a measure of the uncertainty of the growth rate calibration, since the “implied ages” are purely based on a hypothetical assumption, and as such have no defined error.
